# The testosterone-dependent and independent transcriptional networks in the hypothalamus of *Gpr54 *and *Kiss1 *knockout male mice are not fully equivalent

**DOI:** 10.1186/1471-2164-12-209

**Published:** 2011-04-28

**Authors:** Leah M Prentice, Xavier d'Anglemont de Tassigny, Steven McKinney, Teresa Ruiz de Algara, Damian Yap, Gulisa Turashvili, Steven Poon, Margaret Sutcliffe, Pat Allard, Angela Burleigh, John Fee, David G Huntsman, William H Colledge, Samuel AJ Aparicio

**Affiliations:** 1 Molecular Oncology and Breast Cancer Program, British Columbia Cancer Research Centre, 675 West 10th Avenue, Vancouver, BC, V5Z 1L3, Canada; 2 Department of Pathology, University of British Columbia, Vancouver, BC, Canada; 3 British Columbia Cancer Agency, 600 West 10th Avenue, Vancouver, BC, V5Z 4E6, Canada; 4 Genetic Pathology Evaluation Centre of the Prostate Centre, Department of Pathology, Vancouver Coastal Health Research Institute, Vancouver, BC, Canada; 5 Reproductive Physiology Group, Department of Physiology, Development, and Neuroscience, University of Cambridge, Downing Street, Cambridge,CB2 3EG, UK

## Abstract

**Background:**

Humans and mice with loss of function mutations in GPR54 (KISS1R) or kisspeptin do not progress through puberty, caused by a failure to release GnRH. The transcriptional networks regulated by these proteins in the hypothalamus have yet to be explored by genome-wide methods.

**Results:**

We show here, using 1 million exon mouse arrays (Exon 1.0 Affymetrix) and quantitative polymerase chain reaction (QPCR) validation to analyse microdissected hypothalamic tissue from *Gpr54 *and *Kiss1 *knockout mice, the extent of transcriptional regulation in the hypothalamus. The sensitivity to detect important transcript differences in microdissected RNA was confirmed by the observation of counter-regulation of *Kiss1 *expression in *Gpr54 *knockouts and confirmed by immunohistochemistry (IHC). Since *Gpr54 *and *Kiss1 *knockout animals are effectively pre-pubertal with low testosterone (T) levels, we also determined which of the validated transcripts were T-responsive and which varied according to genotype alone. We observed four types of transcriptional regulation (i) genotype only dependent regulation, (ii) T only dependent regulation, (iii) genotype and T-dependent regulation with interaction between these variables, (iv) genotype and T-dependent regulation with no interaction between these variables. The results implicate for the first time several transcription factors (e.g. *Npas4, Esr2)*, proteases (*Klk1b22*), and the orphan 10-transmembrane transporter TMEM144 in the biology of GPR54/kisspeptin function in the hypothalamus. We show for the neuronal activity regulated transcription factor NPAS4, that distinct protein over-expression is seen in the hypothalamus and hippocampus in *Gpr54 *knockout mice. This links for the first time the hypothalamic-gonadal axis with this important regulator of inhibitory synapse formation. Similarly we confirm TMEM144 up-regulation in the hypothalamus by RNA in situ hybridization and western blot.

**Conclusions:**

Taken together, global transcriptional profiling shows that loss of GPR54 and kisspeptin are not fully equivalent in the mouse hypothalamus.

## Background

In 2003 it was discovered that the kisspeptin activated GPR54 receptor is required for maturation and activity of the hypothalamic-pituitary-gonadal axis [1,2]. It has been established subsequently that kisspeptin (Kp) binding to GPR54 releases gonadotropin releasing hormone (GnRH) from the hypothalamus to stimulate the pituitary-gonadal axis in a multitude of species including humans [3-15]. In mice, GnRH neurons express *Gpr54 *from birth and more GnRH neurons acquire expression during pubertal development [16]. In rodents, Kp is expressed by neurons in the arcuate nucleus (ARC) of the hypothalamus and also in the rostral periventricular area of the 3rd ventricle (RP3V) which includes the anteroventral periventricular nucleus (AVPV). Rising estrogen levels have a positive feedback effect on Kp expression in the AVPV [17,18] to initiate the LH surge required for ovulation in females [19-21]. Conversely, estrogen and testosterone have a negative feedback effect on Kp expression in the ARC that is regulated through the estrogen receptor alpha (ERα) or through the androgen receptor (AR) respectively [17-19,21,22].

Despite the critical importance of GPR54/kisspeptin in mammalian fertility, little is known of the upstream and downstream gene regulatory networks for kisspeptin signaling in the hypothalamus. The feedback relationships between GPR54 and kisspeptin are incompletely understood. To address the question of which genes are co-dependent on GPR54 and kisspeptin and whether the transcriptional networks of GPR54 deficient mice and kisspeptin deficient mice are equivalent, we have taken advantage of the knockout mice we generated during the initial discovery of the GPR54-kisspeptin axis. *Gpr54 *and *Kiss1 *knockout mice model the hypogonadotropic hypogonadism (HH) found in humans with *GPR54 *mutations [2,10,23]. Although the neuronal inputs and projections of GPR54 and kisspeptin are barely described, the hypothalamus is clearly a key site of interest, since the action of kisspeptin on neurons co-expressing GPR54 and GnRH are key to the function of the axis. We therefore chose to focus on determining the hypothalamic transcription network, initially scanning the transcriptome with Affymetrix Exon 1.0 arrays. These exon arrays contain probes for all of the known and predicted gene exon sequences in the mouse genome, thus representing a near complete, transcriptome profiling method.

Using micro-dissected hypothalamic tissues to isolate RNA from our mutant mice, we first set out to define the transcriptional differences between the knockout mice. Since the GPR54-kisspeptin axis is subject to hormonal feedback and the knockout mice are pre-pubertal, we also tested the hormonal dependence/independence of each differentially expressed transcript. To avoid variation in gene expression due to fluctuations in the levels of circulating hormones during the female estrous cycle, only male mice and testosterone (T) exposure were used in this study. We utilized a study designed to define purely T-regulated transcription, purely genotype-dependent transcription and transcription dependent on both T and genotype, with interaction or no interaction between these variables.

## Results

### Microarray identification of deregulated transcripts from *Gpr54 *and *Kiss1 *knockout mice hypothalami

**Figure 1:**
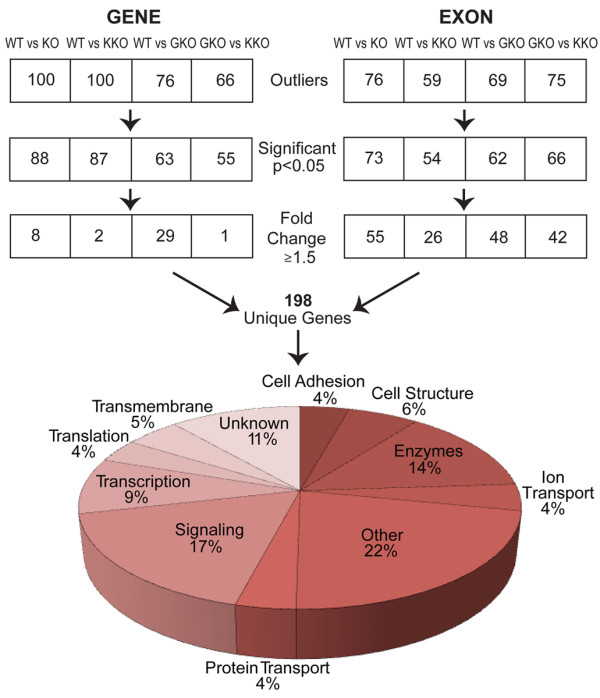
**Classification of 198 differentially expressed genes in the discovery set**. The figure shows the number of significant genes identified at each step of the microarray analysis procedure with subsequent filtering steps for statistical significance and fold change expression differences. WT vs KO represents a joint comparison of all data, whereas WT vs GKO and KKO represent separated analyses. The numbers of genes falling through the analysis are shown separately for gene level and exon level summarization. The pie chart shows the grouping of molecular functions for the 198 gene set.

We first isolated RNA from micro-dissected hypothalamic tissue of knockout and wild-type mice and hybridized this to Exon 1.0 arrays (methods). We compared quantile normalized probe intensity values from Affymetrix whole mouse exon array chips where hybridization was performed with wild-type (WT), *Gpr54 *knockout (GKO) or *Kiss1 *knockout (KKO) hypothalamic RNA. Affymetrix results were compared between genotypes, specifically gene expression of all wild-type mice were grouped together and compared with all knockout mice grouped together (WT vs KO) or with *Kiss1 *knockout mice alone (WT vs KKO) or *Gpr54 *knockout mice alone (WT vs GKO). Additionally, *Gpr54 *knockout mice were compared with *Kiss1 *knockout mice (GKO vs KKO) to give a total of four groups (Figure 1). Gene level and exon level summarization was used in the comparisons and from each of these we selected genes showing a *p*-value < 0.05 and an expression fold difference of 1.5 or greater, as candidates for further analysis (yellow highlighted regions in the volcano plots shown in Additional file 1).

This analysis yielded 198 unique genes, putatively exhibiting differential transcription between wild-type mice and/or either knockout (Figure 1). Several global features of the transcription network were apparent in these data. First, we analyzed the functional groupings of genes and noted that signaling molecules and enzymes were the two largest biochemical classes (Figure 1). Next, we examined possible network overlaps by analyzing separately for the GKO and KKO differential gene lists, the possible network connectivity using a curated database of functional interactions, as previously described (Ingenuity pathways, http://www.ingenuity.com, methods). Interestingly both the GKO (Additional files 2 and 3) and KKO (Additional files 3 and 4) networks show apparently common nodes of signaling via HNF4a and growth factor signaling pathways, however many differences are also seen in the putative networks, suggesting that the effects of *Kiss1 *knockout may not be fully equivalent to *Gpr54 *knockout. Consistent with this notion, we noticed fewer statistically significant gene level (2 vs 29, Additional file 5: Table S1) and exon level (26 vs 49) transcription differences for KKO mice compared with GKO. To explore this possibility further we decided to validate a subset of the most pronounced array expression differences by QPCR. Genes showing transcription differences were prioritized in the following order: strength of expression, consistency of differences between knockouts, known or suspected roles in hormonal regulation, and finally the availability of quenching QPCR probes for low density 384 well QPCR plate analysis. Within these criteria, all transcription factors, transmembrane receptors, and signaling molecules that were available were included, resulting in a total of 72 genes (Additional file 6: Table S2, non-bolded genes). QPCR control probe sets for 18S rRNA and *Gapdh *were included in each array as loading controls and we also selected a number of genes of interest (Additional file 6: Table S2, bold genes) based on their known role in gonadotropic signaling, that were not differentially expressed by microarray analysis. Thus a total of 95 genes were re-validated by microplate QPCR analysis (Additional file 6: Table S2).

We used a 384 well plate QPCR array to assay each gene at least 3 times per sample (technical replicates) and 3 biological replicates of RNA prepared from micro-dissected hypothalamic tissue from wild-type, GKO and KKO mice. After analysis of the count (Ct) values by a linear mixed effects model, genes showed statistically significant differential expression in one of the groups, 7 of which had a fold change ≥ 1.5; two of these were genes of interest (*Esr1, Mmp2*) and one was a control (*Kiss1*; Table 1). All genes with a statistically significant fold change were carried forward for further analysis.

**Table 1:** Discovery set genes showing QPCR validated transcriptional differences between WT and KO (*Gpr54 or Kiss1*)

	GKO vs WT		KKO vs WT	
**Gene Target**	**FC (95%CI)**	**Direction**	**FC (95%CI)**	**Direction**

*Abca8a*	1.85 (1.33-2.59)	Up	1.97 (1.33-2.90)	Up
*Esr1*	1.62 (1.37-1.92)	Up	1.54 (1.32-1.78)	Up
*Kiss1*	14.03 (10.86-18.11)	Up	N.A.	N.A.
*Mmp2*	1.51 (1.18-1.94)	Up	n.s.	n.s.
*Npas4*	2.60 (2.09-3.25)	Up	n.s.	n.s.
*Lrdd*	n.s.	n.s.	1.80 (1.29-2.51)	Up
*Tmem144*	n.s.	n.s.	2.61 (2.21-3.08)	Up

FC = fold change, CI = confidence interval, n.s. = not significant, N.A. = not applicable. GKO = GPR54 knockout, KKO = Kisspeptin knockout.

### Analysis of hormone-dependent and independent hypothalamic gene transcription in *Gpr54 *and *Kiss1 *knockout mice

Array expression analysis of micro-dissected hypothalamic samples revealed significant differential transcription between genotypes. However, since GKO and KKO mice fail to undergo puberty an unknown number of these transcriptional differences could be explained by the hormonal milieu. To address this, we tested which of these genes exhibited hormonally responsive transcription, by measuring transcriptional differences in the presence or absence of testosterone (T). WT, GKO and KKO male mice were castrated and implanted with either a sham silastic capsule, or a T containing capsule (characteristics of the mice used in Additional file 7: Table S4). After 4 weeks exposure, the mice were killed, the hypothalamus micro-dissected, and the RNA extracted. To confirm exposure to T, we measured plasma free testosterone; KKO mean free testosterone 4.0 pg/ml (SD ± 1.5), GKO mean free testosterone 5.8 pg/ml (SD ± 2.7), and WT mean testosterone 5.3 pg/ml (SD ± 1.5) with zero free testosterone in the castrate/empty silastic implant group. Each gene was assayed with at least 4 technical replicates and 3 biological replicates. To reduce the risk of loss of data due to genotype or hormonal effect on loading controls, we used two independent loading controls, 18S rRNA and *Gapdh*. The data were analyzed separately using a linear mixed effects model (methods). We analyzed GKO and KKO mice separately in a 2 × 2 × 2 design, considering gene (test, loading control), treatment (T+, T-) and genotype (WT, GKO or KKO) as categorical variables. Tables 2, 3, 4, and 5 summarize the statistically significant (p < 0.05), greater than 1.5 fold expression difference, model results for four classes of variation: (i) purely hormone-dependent transcription (ii) purely genotype-dependent transcription (iii) hormone and genotype-dependent transcription but with no interaction between these variables and (iv) hormone and genotype-dependent transcription, with co-dependence (interaction) between these variables. Since two loading controls were used, these are also reported separately as they were treated independently in the analysis. Small differences with loading controls were noted, resulting in differences of interaction between genotype and hormone responsiveness for a few transcripts at statistical boundaries of significance. Nevertheless, the direction and magnitude of effects were consistent for all genes, with both control probes. We noted fewer statistically significant transcript regulation effects in the KKO mice, mirroring the initial observations from microarrays. Nevertheless, several genes (eg *Klk1b22, Gnrhr, Tmem144*) were regulated in common with GKO mice, albeit with effects of different magnitude. Several of the statistically significant QPCR results are of small absolute magnitude and considering the variances of the loading controls between genotypes and treatments, we suggest that interpretation of differences of less than 1.5 fold may not be meaningful (full tables in Additional file 8). We further considered only changes of > 1.5 fold.

**Table 2:** Pure hormonal (T) responsive genes

		Hormonal Effect		
**Genotype**	**Gene Target**	**FC (95%CI)**	**Direction**	***p*-value**

GKO	*Abca8a*	1.57(1.33-1.87)	Down	2.83E-05
GKO	*Klk1b22*	2.16(1.43-3.25)	Down	0.0042
KKO	*Gnrhr*	2.33(1.63-3.33)	Down	0.0009

FC = fold change, CI = confidence interval, Dir = direction of change, relative to T. GKO = GPR54 knockout, KKO = Kisspeptin knockout.

**Table 3:** Pure genotype effect genes

		Genotype Effect		
**Genotype**	**Gene Target**	**FC (95%CI)**	**Direction**	***p*-value**

GKO	*Esr2*	1.63(1.46-1.82)	Down	4.10E-10
GKO	*Hhip*	1.63(1.46-1.82)	Down	4.68E-10
GKO	*Lhcgr*	1.61(1.30-1.99)	Down	0.0042
GKO	*Npas4*	1.65(1.45-1.87)	Down	2.06E-08
KKO	*Tmem144*	1.50(1.30-1.73)	Up	6.12E-06

FC = fold change, CI = confidence interval, Dir = direction of change, relative to WT, GKO = GPR54 knockout, KKO = Kisspeptin knockout.

**Table 4:** Genes showing hormonal and genotype transcriptional differences, but without co-dependence

		Genotype Effect		Hormonal Effect		
**Genotype**	**Gene Target**	**FC (95%CI)**	**Direction**	**FC (95%CI)**	**Direction**	***p*-value**

GKO	*Abca8a*	1.35(1.15-1.58)	Down	1.58(1.35-1.85)	Down	1.39E-07
GKO	*Ddx3y*	1.52(1.31-1.77)	Down	1.21(1.04-1.40)	Down	1.52E-05
GKO	*Kiss1*	1.96(1.64-2.35)	Up	12.15(10.15-14.55)	Down	4.29E-46
GKO	*Tmem144*	1.86(1.60-2.16)	Down	1.28(1.10-1.48)	Down	1.51E-10
KKO	*Abca8a*	1.21(1.06-1.37)	Up	1.64(1.45-1.86)	Down	1.75E-09

FC = fold change, CI = confidence interval, Dir = direction of change relative to WT (genotype panel) and T (hormone panel). GKO = GPR54 knockout, KKO = Kisspeptin knockout

**Table 5:** Genes showing hormonal and genotype transcriptional differences, with co-dependence

		Genotype Effect within Hormonal Group				Hormonal Effect within Genotype				
			
		No testosterone		Testosterone		Wild-type		Knockout		
**Genotype**	**Gene **	**FC (95%CI)**	**Dir**	**FC (95%CI)**	**Dir**	**FC (95%CI)**	**Dir**	**FC (95%CI)**	**Dir**	***p*-value**

GKO	*Acsm3*	1.26(1.01-1.57)	Up	2.28(1.84-2.83)	Down	1.93(1.54-2.42)	Up	1.49(1.21-1.84)	Down	2.02E-08
GKO	*Ar*	1.23(1.09-1.38)	Down	1.56(1.39-1.75)	Down	1.13(1.01-1.27)	Up	1.12(1.00-1.25)	Down	1.02E-08
GKO	*Esr1*	1.08(0.96-1.21)	Down	1.31(1.17-1.47)	Down	1.28(1.14-1.43)	Down	1.55(1.39-1.74)	Down	1.36E-11
GKO	*Gnrhr*	1.28(0.78-2.12)	Down	3.20(1.70-6.03)	Up	2.29(1.37-3.82)	Down	1.80(0.96-3.35)	Up	1.80E-03
GKO	*Kiss1*	1.95(1.58-2.42)	Up	1.09(0.88-1.35)	Up	9.12(7.36-11.29)	Down	16.34(13.19-20.23)	Down	5.71E-52
GKO	*Klk1b22*	1.25(0.71-2.20)	Up	2.70(1.61-4.52)	Down	1.08(0.61-1.91)	Down	3.65(2.17-6.12)	Down	0.0004
GKO	*Mmp9*	1.11(0.91-1.35)	Up	1.70(1.40-2.07)	Up	1.11(0.92-1.35)	Up	1.71(1.41-2.08)	Up	2.69E-07
GKO	*Six2*	1.05(0.73-1.50)	Down	2.22(1.55-3.18)	Up	1.78(1.24-2.55)	Down	1.30(0.91-1.87)	Up	0.0022
GKO	*Tec*	2.48(1.95-3.14)	Down	1.75(1.38-2.22)	Down	1.63(1.29-2.07)	Down	1.16(0.91-1.46)	Down	2.03E-13
GKO	*Txnip*	1.04(0.80-1.34)	Down	1.81(1.40-2.35)	Up	1.85(1.43-2.39)	Down	1.02(0.79-1.32)	Up	8.79E-05
KKO	*Acsm3*	1.23(1.03-1.48)	Up	1.27(1.03-1.56)	Down	1.86(1.53-2.26)	Up	1.19(0.98-1.45)	Up	9.80E-06
KKO	*Klk1b22*	1.30(0.92-1.84)	Down	4.30(3.01-6.15)	Down	1.34(0.97-1.84)	Down	4.43(3.01-6.51)	Down	1.75E-09
KKO	*Mmp9*	1.03(0.68-1.56)	Down	3.75(2.44-5.75)	Down	1.08(0.74-1.59)	Down	3.93(2.48-6.22)	Down	0.0127
KKO	*Six2*	1.08(0.84-1.40)	Up	1.99(1.54-2.57)	Up	1.60(1.24-2.07)	Down	1.15(0.89-1.49)	Up	1.14E-05
KKO	*Tec*	1.07(0.86-1.32)	Down	1.72(1.39-2.12)	Up	1.63(1.32-2.02)	Down	1.12(0.91-1.38)	Up	1.82E-05
KKO	*Txnip*	1.16(1.00-1.34)	Down	1.22(1.06-1.41)	Up	1.66(1.44-1.92)	Down	1.18(1.02-1.36)	Down	3.01E-08

FC = fold change, CI = confidence interval, Dir = Direction relative to WT (genotype subpanel) or T (hormone subpanel). GKO = GPR54 knockout, KKO = Kisspeptin knockout.

**Figure 2:**
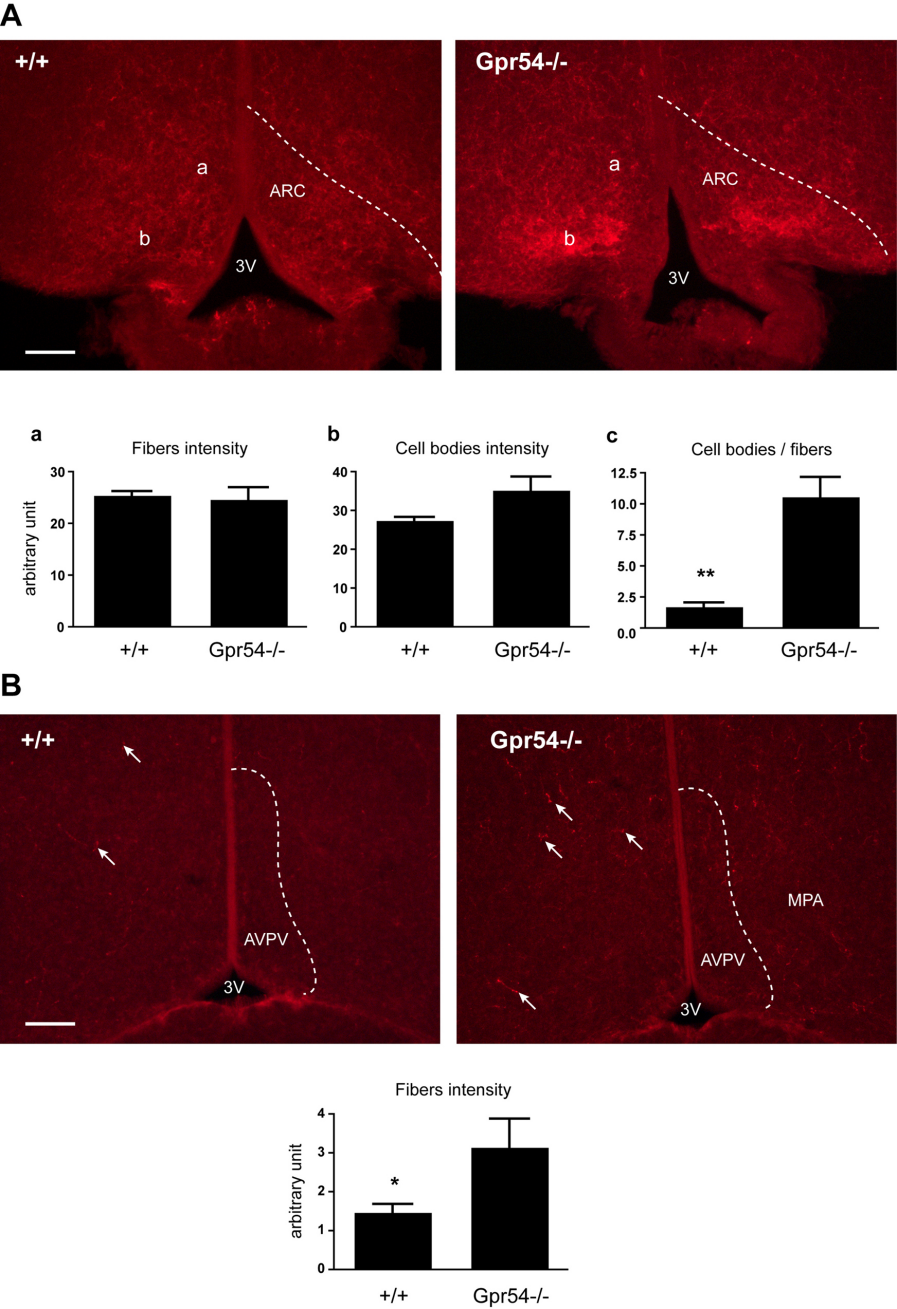
**Kisspeptin immunostaining in WT and GKO mice brains**. (A) Representative photomicrograph of immunocytochemical staining for kisspeptin (red) in coronal brain sections of the arcuate nucleus (ARC) from wild type (WT) and *Gpr54 *knockout (GKO) adult male mice. Bargraphs represent the fiber density in the dorsal ARC (a); the cell body density (b); and the ratio of cell body intensity/fiber intensity (c = b/a). Note the significantly higher cell body/fiber ratio in the GKO brain. (B) Immunocytochemical staining for kisspeptin (red) in coronal brain sections of the anteroventral periventricular nucleus (AVPV). Arrows indicate kisspeptin-positive fibers in the AVPV and in the medial preoptic area (MPA). The bargraph at the bottom represents density of fibers calculated in the AVPV. * *p *< 0.05, * *p *< 0.01, unpaired t-test. 3 V: third ventricle. Scale bar: 200 μm.

The greatest transcript level regulation was observed for *Kiss1 *(Tables 1, 4), measured in GKO mice which showed strong hormonal regulation (approximately 12 fold down-regulated with T) and moderate genotype dependence (approximately 2 fold up-regulated in the GKO animals). With the 18S rRNA loading control, the regulation shows a weak interaction whereas with the *Gapdh *loading control, hormonal regulation, and genotype regulation vary symmetrically and thus no interaction is reported. The hormonal effect of *Kiss1 *expression in the male mouse has already been shown [22], here, we validated the *Kiss1 *up-regulation in hormonally untreated intact male GKO mice by IHC using a kisspeptin antibody that has been previously characterised by our group and others [20]. Kisspeptin immunoreactivity was examined in coronal sections of the regions containing the anteroventral periventricular nucleus (AVPV) and the arcuate nucleus (ARC) from four WT and four GKO adult male mice (Figure 2). In terms of kisspeptin fiber distribution, fibers were found in large numbers in the ARC in both WT and GKO mice, and with virtually no cell bodies observed in the AVPV region. Kisspeptin neuron cell bodies, observed by an intense staining in the dorsal part of the ARC, are significantly more abundant in the GKO ARC than the WT ARC (*p *< 0.01). Kisspeptin fibers have a similar intensity between the two groups. Although kisspeptin immunoreactivity is modest in the AVPV region, we found more kisspeptin fibers in GKO than WT in this region (*p *< 0.05).

The second largest effects were found for the *Klk1b22 *gene (also known as β-NGF-endopeptidase) showing a significant hormonal influence (Tables 2, 5) and 3-4 fold regulation. As expected, the GnRH receptor (*Gnrhr*) also exhibited strong hormonal regulation and a weak genotype effect but with some interaction (the hormone effect is relatively stronger in the KO than the WT mice, compatible with a priming effect), however with large variances of expression.

In KKO mice, only *Gnrhr *showed a pure hormonal regulation of its transcript and only *Tmem144 *showed a purely genotype-dependent regulation. The latter was consistent in direction and magnitude with the levels measured in the non-castrate group of animals used for discovery (Table 1), emphasizing this to be a robust difference. Among the genotype and hormone variant changes, *Klk1b22 *showed the greatest differences, more than 4 fold, with testosterone exposure and this effect was significantly greater (p < 0.05) in the knockout mice (both alleles) than wild-type.

In GKO mice, *Klk1b22 *showed a hormone only effect (down-regulation) with the *Gapdh *control, but a hormone effect with genotype interaction using the 18S rRNA loading control. *Gnrhr *shows a strong hormonal effect with a genotype interaction - down-regulation in the wild-type mice and up-regulation in the knockout mice in the presence of testosterone. *Tmem144 *shows a strong genotype effect in the GKO mice (down-regulated where *Kiss1 *is overexpressed), but in the opposite direction to KKO mice (up-regulated in the absence of *Kiss1*), and with an additional marginal hormone effect. We confirmed that *Tmem144 *is significantly up-regulated in the KKO hypothalamus of the intact male mouse as compared to WT by RNA *in situ *hybridization (*p *< 0.001, Additional file 9). Both KKO and WT antisense probes had significantly more intense staining as compared to their respective sense probe control (*p *< 0.001), and there was not a significant difference between the two control sense probes (*p *= 0.632).

To determine whether the up-regulation of the *Tmem144 *gene expression in KKO mice observed by RT-PCR and *in situ *hybridization existed at the protein level, we performed a series of western blot experiments (Additional file 10). Hypothalami dissected from WT and KKO intact male mice were separated into the anterior and posterior halves. Immunoblot analysis with an anti-TMEM144 antibody revealed a band at the expected size of 39 kDa, and other non-specific bands at various sizes. Quantification of TMEM144 protein levels (band at 39 kDa), by normalizing with β-tubulin protein levels, showed significantly higher TMEM144 protein levels in KKO anterior hypothalamus compared to WT (*p *= 0.034, Student's t-test). In the posterior hypothalamus, TMEM144 protein levels were higher in KKO than WT, although not significantly (*p *= 0.131).

Genotype only effects were seen in GKO mice at > 1.5 fold but less than 2 fold for ERβ (*Esr2*), *Hhip, Lhcgr*, and *Npas4*. We also noticed several matrix metalloproteinase (MMP) family members (*Mmp2, Mmp9, Mmp28*) showing mixed hormonal and genotype effects, but for *Mmp2 *at a fold change less than 1.5. *Mmp2 *was down-regulated by 1.4 fold in the GKO mice compared to WT and had an independent hormonal effect shown by a 1.2 fold decrease in gene expression when testosterone treatment was compared to the empty silastic control. *Mmp28 *had a genotype only effect in the GKO mice and was down-regulated by 1.5 fold when compared to WT (Additional file 8: Table S10). We did not analyze in a joint statistical comparison the GKO and KKO transcript variations from the discovery experiment with the GKO T- and KKO T- animals of the validation experiment, because they are not biologically equivalent (see discussion), since the immature testis may be a source of feedback regulation to the hypothalamus.

**Figure 3:**
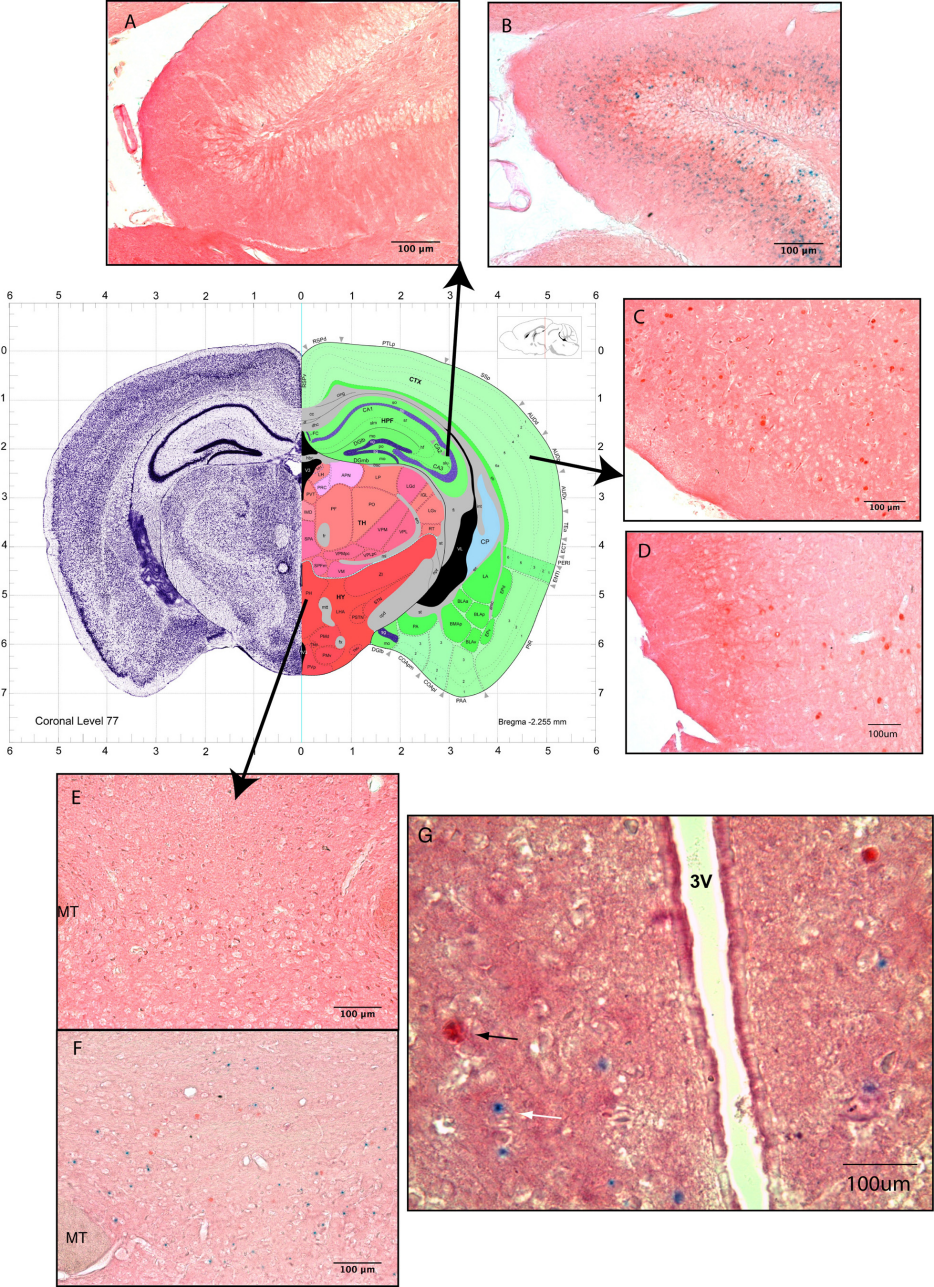
**Localization of NPAS4 in the brain of WT and *Gpr54 *-/- mice**. For clarity the brain regions represented in panels A-F are shown mapped to an equivalent coronal section of the Allen brain atlas, corresponding to coronal level 77 (http://mouse.brain-map.org). The conceptual nuclear organization is shown in the right half of the panel, annotations are available at the brain atlas website. (A) WT mouse hippocampus stained with NPAS4 antibody (B) *Gpr54 *knockout (GKO) hippocampus stained with beta gal (blue cell bodies) and NPAS4 (red-brown). (C) WT cortex stained with NPAS4 antibody (red-brown) (D) GKO cortex stained with beta-gal (blue) for Gpr54 transcription and NPAS4 protein (red-brown) (E) WT posterior hypothalamus stained with NPAS4 antibody (red-brown) (F) GKO posterior hypothalamus stained with beta gal (blue) for Gpr54 transcription and NPAS4 protein (red-brown). (G) a more anterior coronal section (level 71, map not shown here) in GKO showing GPR54 transcription (blue, beta gal) and NPAS4 immunoreactivity in the periventricular hypothalamus. No endogenous beta-gal activity was seen in the wild-type mice in any of the brain regions. MT: mammilothalamic tract. 3 V: third ventricle.

Npas4, which has very recently been described [24] as an important activity-dependent transcription factor regulating inhibitory synapse formation in the GABAergic system, was up-regulated in GKO mice and was the only novel transcript for which IHC grade antibodies were readily available (generous gift of Michael Greenberg). The NPAS4 protein was expressed in cortical cell bodies of WT (44/hpf - 40× high power field) and GKO (26/hpf) mice (Figure 3C,D). NPAS4 immunoreactivity (ir) was also seen in the hippocampus (6/hpf) (Figure 3A, B), posterior hypothalamus (Figure 3E,F) (11/hpf) and periventricular hypothalamus (7/hpf) (Figure 3G) of GKO mice whereas no NPAS4 ir was detectable in WT for any of these regions (0/field) mice. As previously described, we used the *Gpr54 *driven beta-galactosidase activity (Figure 3, blue neuronal cell bodies) to examine co-localisation of Gpr54 and NPAS4, however none of the cell bodies overlapped in expression, indicating that the alteration of *Npas4 *expression in GKO mice is non cell-autonomous with respect to *Gpr54*.

## Discussion

We have assessed gene expression variability amongst *Kiss1 *and *Gpr54 *knockout mice (KKO, GKO) to determine potential gene candidates involved in either direct or indirect regulation of this ligand-receptor pair. Initially an Affymetrix Exon 1.0 ST Array consisting of more than 1 million probe sets (exons) was used to find gene expression differences between the genotypes and littermate wild-type mice. Initial array analysis indicated that while there were many overlapping transcription differences between the alleles, some differences were noted. Putative network analysis showed apparently common nodes in HNF4a and some growth factor pathways, however non-overlap was seen in other nodes. Moreover fewer statistically significant expression differences were noted in the *Kiss1 *knockout mice. We re-validated these transcriptional differences, using QPCR with quenching probes and using two loading control probes. This yielded a set of 72 genes with confirmed transcriptional differences between genotypes, which were supplemented with an additional 23 genes of interest and controls. Since the knockout mice fail to undergo puberty, it was necessary to separate transcripts that may be subject to hormonal regulation, from those that are independent of hormonal regulation. In a second series of experiments we assessed the expression of the 72 array-derived gene panel and 23 genes of interest, in the hypothalamus of castrated mice, treated with or without hormonal implants prior to gene expression comparisons between genotypes. The comparison of T+, T-, and genotype groups within the linear mixed effects model analysis allowed us to observe genotype-dependent variation of hormonal response and genotype-independent hormonal response.

Around 500 GnRH expressing neurons are present in the mouse hypothalamus [25,26]. Considering the results of the testosterone/genotype experiment, the detection of significant changes in *Gnrhr *and *Kiss1 *transcripts indicates that the relevant region of the hypothalamus was sampled and that the method used was capable of picking up transcriptional differences in two genes known to be hormonally regulated. Our data show for the first time, counter regulation of *Kiss1 *transcripts and protein expression in *Gpr54 *knockout mice. This is consistent with previous reports showing that upon interruption of the hypothalamic-pituitary-gonadal axis, *Kiss1 *is elevated in the hypothalamus of rodents [8,22,27], primates [28] and women [29].

It has been established that the anteroventral periventricular nucleus (AVPV) and the arcuate nucleus (ARC) are the key regions in the rodent hypothalamus involved in *Kiss1 *expression [17-19,21,22,30]. We show here for the first time that kisspeptin expression in male GKO mice is localized to the cell bodies in the ARC and absent in the AVPV. This is in accordance with the sexual dimorphism in kisspeptin expression whereby males have far fewer kisspeptin neurons in the AVPV region than females [30]. Additionally, the kisspeptin neuron cell bodies are significantly more abundant in the GKO ARC than the WT while kisspeptin fibers have a similar intensity between the two groups. Although kisspeptin staining was modest in the AVPV region, we found more kisspeptin fibers in GKO than WT. Thus, the up-regulation in *Kiss1 *gene expression in the hypothalamus observed by RT-PCR using the whole hypothalamus actually occurs in the ARC only since there were no more kisspeptin cell bodies observed in the AVPV from GKO compared to WT mice.

Small differences, although statistically significant, should be interpreted with caution and considering the degree of variation seen with the loading control estimates for the samples, we did not consider absolute effects of less than 1.5 fold to be interpretable, even if statistically significant. It should also be noted that especially where interaction effects are seen, genotype and hormone interaction effects may depend upon differences in the hormonal milieu prior to castration, that may have occurred during development and this limitation should be recognized in generalizing the results. For example, regulation of GnRH receptor levels may show a "priming" effect in wild-type castrate mice from prior perinatal sex steroid exposure, compared with knockouts. The reversed direction of hormonal effect for *Gnrhr *between GKO and WT is consistent with such an explanation [31]. As we were able to observe robust regulation of *Gnrhr*, which is predominantly restricted to the 500 or so GnRH expressing neurons in the mouse hypothalamus, this suggests that the study was powered sufficiently to reveal significant commonalities and differences in the transcriptional networks of *Gpr54 *and *Kiss1 *knockout mice.

Interestingly, relatively few large differences in transcription were seen in the initial Affymetrix array experiment, and overall, fewer changes were seen in the *Kiss1 *knockout mice, than in the *Gpr54 *knockout mice. The subsequent detailed QPCR analysis showed this result to be reproducible. Although both genotypes fail to undergo puberty, it has been reported that the kisspeptin loss of function alleles may be less severe than the *Gpr54 *knockout mice [32]. The endogenous basal activity of the GPR54 receptor could explain such a difference [33] as could the existence of an unknown weaker binding peptide ligand for GPR54. This result is of importance for future studies directed at understanding the signaling consequences of GPR54 and kisspeptin activity.

We did not compare directly the control group of castrate -T mice with the intact knockout mice. Although the intact knockout mice are also sexually immature, the intact immature testis may still have active feedback to the hypothalamus and transcription differences would be expected. Indeed, a greater difference in *Kiss1 *expression was seen in the intact GKO mice than the castrate GKO mice. This is consistent with reports showing that *Kiss1 *expression is up-regulated in the hypothalamus of castrated rodents [12,22]. We also noticed opposite direction of regulation for the genotype-only transcripts (i.e. for transcripts where T exposure had no effect) *Npas4 *and *Abca8a *between the first and second groups.

The proteolytic pre-processing enzymes responsible for generating active kisspeptins in the hypothalamus are unknown. It has been shown that Kp can down-regulate MMP9 expression [34]. We observed that *Mmp9 *is up-regulated when there is *Kiss1 *loss in the KKO mice and the expected down-regulation is observed when there is gain of *Kiss1 *expression, as we see in the GKO mice, however these observations are specific to the hormonally treated group for both mutants. Similarly MMP2 has been shown to be down-regulated by Kp [35] and in our study *Mmp2 *is down-regulated in the highly expressing *Kiss1 *GKO mice, although at a fold change less than 1.5. Additionally, reciprocal up-regulation is absent in the KKO mice. Although it has been demonstrated that MMP9/2 can cleave and inactivate Kp, the precise relationship between the MMPs and Kp remains to be elucidated. *Mmp28 *exhibited a 1.5 fold genotype only regulation, suggesting it as another potential regulator of kisspeptin. Although all the MMPs were consistent in showing statistically significant transcription changes in our analysis, the extent of regulation was less than 1.5 fold for *Mmp2 *and hence these results should be interpreted with caution.

Beyond the matrix metalloproteases, we observed *Klk1b22 *to be strongly regulated in the KKO and GKO mice. *Klk1b22*, also known as β-nerve growth factor (NGF)-endopeptidase [36] is a member of the kallikrein gene cluster that has been expanded by tandem gene duplication. The predicted activity pattern for *Klk1b22 *is cleavage at Arg-Ser residues, an arrangement found within mouse pre-pro kisspeptin that would result in a 52 amino acid peptide, similar to the Kp54 seen in humans. However, *Klk1b22 *does not have an ortholog in the human genome, although several very close paralogs exist in the duplicated kallikreins. The peptide cleavage sequence is not conserved in the human pre-pro kisspeptin, rather, the bond that is cleaved in the human sequence is Arg-Gly to create the N-terminus of Kp-54 [37,38]. Our data nevertheless suggest that this kallikrein family of peptidases is worthy of future exploration as kisspeptin processing enzymes, especially since we show strong evidence of regulation in response to sex steroids.

*Tmem144 *was the only transcript that was consistently up-regulated in the KKO mice as compared with WT mice, independent of testosterone exposure and in all groups of KKO mice. We confirmed this up-regulation by RNA *in situ *hybridization and western blot analysis of TMEM144 in the hypothalamus of KKO mice as compared to WT mice. *Tmem144 *encodes an orphan 10-transmembrane family receptor, whose solutes and activities are unknown. This class of transmembrane proteins can transport a wide variety of molecules, ranging from sugars and amino acids to solutes and are generally linked to Ca^2+ ^mediated activation of gene expression. Activation of non-selective cation channels and inhibition of inwardly rectifying potassium channels have been shown to be necessary for Kp to depolarize GnRH neurons [39,40]. It is possible that TMEM144 is somehow implicated in the regulation of kisspeptin expression, its expression was high in the KKO mice lacking *Kiss1 *transcripts and low in the GKO mice where *Kiss1 *is over-expressed, suggestive of a direct or indirect negative regulator of kisspeptin. Future genetic studies of *Tmem144 *will be required to evaluate this possibility.

The transcription factor gene *Npas4 *was up-regulated in the GKO mice with one of the largest fold changes after the *Kiss1 *transcripts yet was down-regulated by 1.7 fold in castrated mice independent of hormonal feedback. This up-regulation in non-castrated mice was confirmed by IHC showing expression of Npas4 in the hypothalamus of *Gpr54*-/- mice while Npas4 expression was mostly absent in the wild-type mice. *Npas4 *has been credited with regulating the development of inhibitory synapses in activating neurons in the hippocampus [24]. Interestingly, *Npas4 *is activated by an influx of calcium [24] and is independent of the MAP kinase pathway [41]. It has been recently reported that Kp-GPR54 signaling in the hypothalamus of rats is also independent of the MAP kinase pathway but can signal with calcium release [39]. Whether the *Npas4 *regulation is direct or an indirect consequence of sexual immaturity, remains to be seen, but its role in calcium dependent activation taken together with our data suggest it may play a role in the gonadotropic axis.

*Estrogen receptor alpha (Esr1) *expression was higher in both GKO and KKO mice compared to sexually mature wild-type males which probably reflects the absence of negative feedback by testosterone in the mutant mice. Consistent with this, *Esr1 *expression was decreased by 1.2 fold in the castrated KKO mice that were give testosterone implants. In contrast, expression of the estrogen receptor beta (*Esr2*) was 1.6 fold lower in the GKO mice independent of testosterone effects. *Esr2 *function in the hypothalamic-pituitary-gonadal axis is currently unknown as hormonal feedback occurs almost entirely through the estrogen receptor alpha [17,19,22] as supported in our study. This is the first report that *Esr2 *may be regulated by Kp and GPR54.

Previous studies have identified genes that show an up-regulation in the hypothalamus during mammalian puberty including *Eap1 *(enhanced at puberty 1) [42], *Oct2 *[43] and *Ttf1 *(thyroid transcription factor 1) [44]. The role of these genes as key regulators of pubertal initiation is not clear however. Disruption of expression of these genes in the hypothalamus delays but does not prevent entry into puberty in contrast to loss of Kp/Gpr54 signaling which is associated with complete loss of puberty. We did not observe any difference in expression of these genes in our analysis suggesting that they may only facilitate puberty rather than act as essential regulators.

## Conclusions

Taken together our results reveal for the first time, using a genome-wide discovery approach, the complex network of gene regulation that is dependent on GPR54 and kisspeptin. We have identified from this network, transcripts whose regulation is strongly dependent on sex-steroid exposure and therefore likely to be a secondary consequence of sexual immaturity in these mutants. Importantly, we have also identified novel transcripts, such as *Tmem144 *whose regulation is independent of sex steroid exposure and are therefore prime candidates for direct involvement in the biology of kisspeptin and GPR54 regulation. Future genetic and biochemical studies will determine the role of these genes in the gonadotropic axis.

## Methods

### Experimental design

Part i) To discover novel gene candidates that may be involved in the Kp-GPR54 signaling pathway, we assessed gene expression differences in the hypothalamus of *Kiss1 *and *Gpr54 *knockout mice (KKO, GKO) compared to wild-type mice (WT). Affymetrix Exon 1.0 ST Arrays sampling approximately 1 million exons, were used to assess gene expression initially. After analysis of the exon array probesets, differentially expressed transcripts were re-validated using QPCR with 384-well low density array (LDA) plates, assayed in an ABI 7900HT real-time PCR device.

Part ii) To account for hormonally regulated transcripts that may be differentially regulated as a consequence of sexual immaturity in the mutant mice yet not directly affected by Kp/GPR54 signaling, a hormonally controlled group of mice were assessed. To ensure equal hormone exposure in all genotypes (GKO, KKO, WT), all mice were castrated prior to treatment. Treatment consisted of either a testosterone implant or an empty silastic control. The hypothalamus was again isolated for assessment of differential gene expression, this time using a smaller QPCR array of 48 genes.

### Animals

*Gpr54 *and *Kiss1 *knockout mice have been previously described [2,10,23]. Male 129S6/Sv/Ev wildtype, 129S6/Sv/Ev *Gpr54- *or 129S6/Sv/Ev *Kiss1- *knockout mice were housed under conditions of 12 hours of light with *ad libitum *access to food and water. The average age of the mice from the first analysis was 60-70 days and 90 days for the second hormonally controlled group. All experimental protocols were performed under the authority of a United Kingdom Home Office Project License and were approved by the Cambridge Animal Ethics Committee.

### Castration and testosterone implants

Adult males were bilaterally castrated under general anaesthesia using Ketamin/Xylasine. Castrated mice were divided into two groups: bilateral castration plus empty implant or bilateral castration plus testosterone implant. Testosterone implants were manually and aseptically prepared in the laboratory using silicone tubing (0.058 inch ID/0.077 inch OD; Dow Corning) filled with crystalline testosterone (T-1500; Sigma Aldrich, UK), and sealed with adhesive silicone type A glue [45]. Implants were inserted subcutaneously at the time of castration. Mice were allowed approximately 3 - 4 weeks for recovery (the first week with paracetamol in the water supply), and killed by CO_2 _exposure. Blood was collected in a heparinized syringe from the inferior vena cava and centrifuged at 1,000 × g for 10 min at 4°C. The plasma supernatant samples were collected and stored at -20°C. The mice characteristics are listed in Additional file 7: Table S4.

### Testosterone assay

Free testosterone was measured in duplicate using an ELISA kit (DB52181; IBL, Hamburg, Germany) with a sensitivity of 0.17 pg/ml and intra- and inter-assay variation coefficients respectively 8.9% and 8.8% [23].

### Hypothalamus dissection for RNA extraction

After removal of the brain, the meninges and optic chiasm were discarded and the hypothalamus was isolated. The external limits for this dissection were: lateral, the external border of the medial preoptic area and more caudally, the lateral borders of the mammillary nucleus; dorsal, 1.5 mm depth; anterior, the anterior limit of the nucleus of the vertical limb of the diagonal band (bregma + 1.34 mm); and posterior, the posterior limit of the mammillary nucleus (bregma - 3.40 mm). The whole hypothalamus contained at least the following main hypothalamic nuclei: arcuate nucleus (ARC), anteroventral periventricular nucleus (AVPV), anterodorsal preoptic nucleus (ADP), magnocellular nucleus (MA), supraoptic (SON) and paraventricular (PVN) nuclei, medial preoptic area (MPO) and the diagonal band of Broca (DBB). Immediately after dissection, hypothalami samples were placed in 600 μl of RNAlater (Applied Biosystems, UK) and kept at 4°C for 24 h and then stored at -20°C until RNA extraction.

### RNA extraction

RNA was extracted using the Qiazol method (Invitrogen, Carlsbad, CA) that is recommended for fatty tissues. Whole hypothalami were removed from RNAlater, with excess reagent blotted off of the tissue with a kimwipe before being placed in 100-200 μl of Qiazol. The hypothalami were then homogenized using a Kontes Pellet Pestle (Fischer Scientific, Ottawa, ON) hand-held homogenizer until a uniform mixture was achieved. The remaining Qiazol up to 1 ml was added to the homogenized mixture and the total homogenate was placed in a phase lock tube to separate out the aqueous phase through centrifugation. RNA was precipitated out of the aqueous phase with an equal volume of isopropanol and pelletted by centrifugation. Seventy percent ethanol was used to wash the pellet that was then air-dried, resuspended into 5.5-11.5 μl of RNase/DNase free water, and heated to 65°C for 10 min. One and a half microliters of RNA was set aside for Agilent analysis and RNA was stored at -80°C.

### Agilent analysis

RNA concentration and integrity (RIN) were determined by the Agilent 2100 bioanalyzer as per manufacturer's recommendations. Briefly, 1 μl of heat denatured RNA mixed with 5 μl of Nano Marker was run on a RNA 6000 NanoChip (Agilent Technologies, Mississauga, ON) already prepared with prefiltered Nano gel matrix mixed with Nano dye concentrate. RNA samples were compared to 1 μl of NanoChip RNA 6000 ladder.

### Affymetrix procedure

Hypothalamic RNA was purified further using the GeneChip Whole Transcript Sense Target Labeling Assay (Affymetrix, Santa Clara, CA) as per manufacturer's recommendations before being hybridized to a GeneChip Mouse Exon 1.0 ST Array (Affymetrix, Santa Clara, CA). One microgram of RNA was used as starting material with all samples having a RIN ranging between 8.2-9.0. Starting RNA was mixed with Control Poly-A-RNA before using the RiboMinus Transcriptome Isolation Kit (Invitrogen, Carlsbad, CA) as per Affymetrix recommendations to reduce the rRNA contamination within the sample. Each batch of RNA run through the RiboMinus kit was run in parallel with mouse liver RNA that had either been rRNA reduced or left unreduced as a comparison for effectiveness of the RiboMinus assay. The rRNA reduced samples and liver controls were then purified and concentrated using the GeneChip In vitro transcription (IVT) cRNA Cleanup Kit and assessed by Agilent analysis. Successfully rRNA reduced samples continued forward to first strand cDNA synthesis using T7-(N)6 primers and SuperScript II enzyme before second strand cDNA synthesis using DNA Polymerase I. Within 10 min of second strand cDNA synthesis, samples were run through the GeneChip Whole Transcript cDNA Amplification Kit before IVT cRNA Cleanup and then assessed for cRNA concentration by NanoDrop analysis (NanoDrop Technologies, Wilmington, DE). Samples with cRNA concentrations equal to or greater than 10 μg in a total volume of 6.5 μl were carried forward to the second cycle of first-strand cDNA synthesis using the GeneChip Whole Transcript cDNA synthesis Kit. Random primers with a deoxyuridine triphosphate (dUTP) component mixed within the deoxynucleotide triphosphates (dNTPs) were used. The single stranded cDNA was then treated with RNase H to hydrolyze any remaining cRNA and run through the cDNA Cleanup Spin Columns as per the GeneChip Sample Cleanup Module. Eluted cDNA was assessed by NanoDrop for sample concentration. Finally, 5.5 μg of single stranded cDNA was fragmented with Uracil DNA Glycosylase (UDG) and APE 1 then labeled with biotin allonamide triphosphate (TdT) using the GeneChip Whole Transcript Terminal Labeling Kit before being hybridized for 17 h to the Mouse 1.0 ST Array chip in the GeneChip Hybridization Oven 640 using the GeneChip Hybridization, Wash and Stain Kit. Before scanning the chip on the GeneChip Scanner 3000 7G, each chip was run through the GeneChip Fluidics Station 450 for washing and staining with Streptavidin Phycoerythrin (SAPE). When the sample yielded a concentration less than required for subsequent steps in the protocol, the process was started over again and resulting volumes amalgamated (and in some instances vacuum centrifuged to increase the concentration) before moving forward to the next step.

### Quantitative real-time polymerase chain reaction (QPCR)

The Applied Biosystems (ABI) 7900HT Fast Real-Time System was used to amplify and detect mouse cDNA transcripts (Applied Biosystems, Foster City, CA). For high throughput detection a Taqman Low Density Array (LDA) Card from ABI consisting of 95 genes including two endogenous control genes (*Gapdh *and 18S rRNA) was custom ordered. To assess the second group of mice independent of hormonal feedback, a second smaller LDA set was ordered consisting of 48 genes. The gene list was similar to that of the first larger LDA and had the same two endogenous controls. The complete list of genes used for both LDAs are in Additional files 2 and 11.

### Network analysis

Analysis was conducted using literature-curated data at http://www.ingenuity.com. A data set containing gene identifiers from the GKO and KKO expression arrays and corresponding expression values was uploaded into in the application. Each identifier was mapped to its corresponding object in Ingenuity's Knowledge Base. All expression values passing initial array significance analysis were included and used. These molecules, called Network Eligible molecules, were overlaid onto a global molecular network developed from information contained in Ingenuity's Knowledge Base. Networks of Network Eligible Molecules were then algorithmically generated based on their connectivity. The top 3 canonical networks with more than 3 molecules were merged into a global network for the GKO and KKO genes (Additional files 2 and 4). For clarity each of the three individual networks is also shown separately (Additional file 3).

### Kisspeptin Immunohistochemistry (IHC)

Four WT and four GKO male mice were deeply anesthetized with pentobarbital (400 mg/kg, i.p.) and perfused transcardially with 20 ml of 4 % paraformaldehyde in 0.1 M phosphate buffer (PB), pH 7.4. Brains were removed and immersed in the same fixative for 1 h at 4°C and stored in PB until sectioned. 40 μm Vibratome (VT1000S; Leica, Wetzlar, Germany) coronal floating sections containing the anteroventral periventricular (AVPV) nucleus and the arcuate nucleus (ARC) regions were incubated for 16 h at 4°C in a polyclonal rabbit anti-kisspeptin-10 antiserum (1:5,000; no. 566, gift from A. Caraty, Tours, France) in Tris-buffered saline (TBS; 0.2 M Tris, 0.15 M sodium chloride) solution containing 0.3% Triton X-100, and 5% normal goat serum. Sections were then washed four times in TBS (10 min/wash) and exposed to Alexa Fluor 568-conjugated anti-rabbit antibody (1:500, Invitrogen) for 1 h at room temperature. After washes, sections were incubated for 5 min with DAPI 10 μg/ml (D9542, Sigma Aldrich) in TBS for nuclear fluorescent staining, washed with TBS, mounted on polysine glass slides and coverslipped in FluorSave (345789, Merck).

Immunofluorescent labelling images were acquired with a fluorescent system (Axia Imager A1 microscope, AxioCam MRc5 camera, Axiovision software; Zeiss). Alexa 568 positive staining was examined with the FS45 filter set (560-630 nm). For illustration purposes, photomontages were prepared with the help of Photoshop Elements 4.0 and Illustrator CS3 (Adobe Systems).

Analysis was undertaken by using digital images acquired with a 10× objective. For the kisspeptin staining analysis in the AVPV region, four images were recorded for each animal under the exact same microscope and software settings. Each image was converted to greyscale format and both hemisections were analyzed. In each hemisection, both background (in the medial preoptic area, distal to the third ventricle) and AVPV intensity were considered. The staining intensity was measured using the histogram function in Photoshop Elements 4.0. Specific intensity in the AVPV was calculated by subtracting the background intensity from the AVPV intensity and was expressed in arbitrary unit. Staining analysis in the ARC region was performed as for the AVPV with four images for each animal but we differentiated the kisspeptin fibers (dorsal ARC) from the kisspeptin cell bodies (ventral ARC). In each hemisection, background intensity was measured in the lower left corner of the image to normalize the data. Kisspeptin fiber intensity and cell body intensity, in the dorsal and ventral parts of the ARC respectively, were calculated by subtracting the background intensity and were expressed in arbitrary unit. The ratio of cell body intensity/fiber intensity was also calculated. Values for each mouse were used to determine mean counts, and these were used to generate means ± SEM values for each group. The comparison between each group was subjected to an unpaired Student's t-test.

### Hypothalamus dissection for protein extraction

Four WT and four KKO male mice were killed by CO_2 _exposure. Brains were removed and the hypothalamus was dissected in two parts: the anterior (AntH) and the posterior hypothalamus (PostH). AntH mainly contains the anteroventral periventricular nucleus (AVPV), the diagonal band of Broca (DBB), the median preoptic nucleus (MnPO), the medial preoptic area (MPA), the medial preoptic nucleus (MPO), and the periventricular nucleus (PeN). PostH mainly contains the arcuate nucleus (ARC), the dorsomedial hypothalamic nucleus (DMH), the lateral hypothalamus (LH), and the ventromedial hypothalamic nucleus (VMH). AntH and PostH tissues were snap frozen in liquid nitrogen and stored at -80°C until protein extraction. Briefly, tissue fragments were dissociated through a 25 G needle in 300 μl of lysis buffer (pH 7.4, 25 mM Tris, 50 mM β-glycerophosphate, 1.5 mM EGTA, 0.5 mM EDTA, 1 mM sodium pyrophosphate, 1 mM sodium orthovanadate, Complete protease inhibitors 1X (Roche), 100 μg/ml PMSF, and 1% Triton X-100. The tissue lysates were cleared by centrifugation at 10,000 g for 15 min and protein contents were determined using the Bradford method (Bio-Rad). Protein concentration of each sample was adjusted with lysis buffer and one volume of loading buffer 4X (80 mM Tris-HCl, 4% SDS, 40% glycerol, 10% β-mercaptoethanol, and bromophenol blue, pH 6.8) was added to 3 volumes of protein lysate. Finally, samples were boiled for 5 min before storage at -80°C until use.

### Western blot analysis

Samples were re-boiled for 5 min after thawing and 30 μg of protein per sample were electrophoresed for about 80 min at 125 V in 10% SDS-polyacrylamide gels using a Bio-Rad Mini-Protean system. After size fractionation, the proteins were transferred onto polyvinylidene difluoride 0.45 μm pore-size membranes (Immobilion-P, Millipore) using a semi-dry blotting apparatus (Atto, Japan) for 75 min at room temperature (RT). Blots were blocked for 1 h in TBS with 0.05% Tween 20 (TBST) and 5% non-fat milk at RT, incubated overnight at 4°C with a rabbit polyclonal anti-TMEM144 primary antibody (SAB2102456, Sigma) at 1 μg/ml dilution. After incubation with primary antibody, membranes were washed four times with TBST before being exposed to HRP-conjugated secondary anti-rabbit IgG diluted in 5% non-fat milk TBST for 1 h at RT. The immunoreactions were revealed with enhanced chemiluminescence (RPN2132, GE Healthcare) and detected with an X-ray film (Konica Minolta). Membranes were then stripped in a stripping solution (62.5 mM Tris-HCl, 2% SDS, pH 6.7, and 100 mM β-mercaptoethanol) for 30 min with gentle rocking at 65°C. Membranes were washed and incubated with HRP-conjugated secondary anti-rabbit IgG antibody was used to verify that all former immunoreactivity was successfully stripped. Membranes were re-probed with a mouse monoclonal anti-β-tubulin 1:10,000 under the same conditions as described above for the anti-TMEM144 antibody and detected with a HRP-conjugated secondary anti-mouse IgG 1:2000.

Analysis was undertaken by using digital images acquired with a scanner Epson Perfection 1260 and processed by Photoshop Elements 4.0. The intensity of each band for TMEM144 and β-tubulin was measured using the histogram function in Photoshop and the relative content of TMEM144 was normalized by calculating the ratio TMEM144 intensity/β-tubulin for each sample. Values for each mouse were used to determine mean counts, and these were used to generate means ± SEM values for each group. The comparison between each group was subjected to an unpaired Student's t-test.

### NPAS4 immunohistochemistry (IHC)

Mice (three of each genotype) were perfused with 4.0% paraformaldehyde/PBS prior to brain dissection, which were then immersed in 20% sucrose overnight. The brains were frozen in OCT and cryosectioned at 20 microns. Sections were placed onto positively charged slides and kept at -80°C. Slides were then hydrated with PBS briefly prior to β-galactosidase treatment overnight [10]. Slides were washed with PBS then incubated with 4% paraformaldehyde for 10 minutes, then washed with PBS followed by a 0.03% hydrogen peroxide treatment for 10 minutes. After a PBS wash slides were incubated with serum-free protein block (Dako) for 2 hours then incubated overnight at 4°C with 1:500 NPAS4 antibody generously donated by Michael Greenberg's laboratory [24]. Secondary antibody was 1:100 Goat anti-Rabbit HRP (Dako) for 2 hours. Reactivity was visualized using DAB chromogenic solution, slides were mounted in 50% PBS and 50% glycerol and coverslipped. At least two brains were examined for each genotype studied. Microscopy was conducted using a Zeiss Axioscope, with phase contrast.

### RNA *in situ *hybridization (ISH)

The frozen brain sections were prepared as for the NPAS4 IHC except at a thickness of 10 microns for two mice of each genotype; WT and KKO. The ISH procedure was based on a protocol developed by Dijkman et al. available through the Roche website (http://www.roche-applied-science.com/PROD_INF/MANUALS/InSitu/InSi_toc.htm). Specifically, slides were heated at 50°C for three minutes and allowed to air dry at RT for 30 minutes. They were then treated with chloroform for 5 minutes and allowed to dry for 5 minutes. The slides were then treated with 4% paraformaldehyde in PBS for 7 minutes then washed in PBS, followed by washes in 2 × SSC. The prehybridization step used 100 μl of hybridization buffer (4 × SSC, 10% dextran sulfate, 1X Denhardt's solution (Sigma-Aldrich) 2 mM EDTA, 50% deionized formamide, 500 μg/ml salmon sperm DNA) at 37°C for 1 hour. The sense and antisense probes were added to the hybridization buffer at a concentration of 200 ng/l and slides were incubated with the probes in a humidified chamber overnight at 37°C.

The probes were created by PCR amplification using cDNA from a WT mouse hypothalami and PCR primers that included the Sp6 and T7 promoter sequences for RNA transcription. The sense primer sequence was ATT TAG GTG ACA CTA TAG ATG AGC AGC AAT GCA ACA GAC and the antisense primer sequence was TAA TAC GAC TCA CTA TAG GGC GTG AGC GCA CTT ACT ACT GA. The PCR product produced the expected single 461 bp band on gel electrophoresis and was Sanger sequenced prior to gel extraction to confirm the correct Tmem144 sequence. The Roche Digoxigenin (DIG) RNA Labeling Kit (SP6/T7) was used to create the DIG label sense and antisense probes as per manufacturer's recommendation.

After probe hybridization the slides were washed with 2 × SSC followed by a 60% formamide in 0.2 × SSC wash both at 37°C. There were final washes with 2 × SSC at RT and with 100 mM Tris-HCl, 150 mM NaCl (pH 7.5) prior to probe detection. The Roche DIG Nucleic Acid Detection Kit (NBT/BCIP) was used to visualize the ISH probes. Specifically, the sections were incubated for 30 minutes at RT with blocking buffer (100 mM Trish-HCl, 150 mM NaCl used to dilute 10X blocking reagent to 1X). The alkaline phosphatase-conjugated sheep anti-DIG was diluted to 1:200 in the blocking buffer and used to incubate the slides for 2 hours at RT. After incubation the slides were washed with 100 mM Trish-HCl, 150 mM NaCl and incubated for 10 minutes with detection buffer (100 nM Tris-HCl, 100 mM NaCl, 50 mM MgCl_2_). Slides were incubated overnight with detection buffer containing 0.18 mg/ml 5-bromo-4-chloro-3-indolyl-phosphate, 0.34 mg/ml nitroblue tetrazolium chloride (Roche kit) and 240 μg/ml levamisole at RT. After a distilled water wash the slides were counterstained with 1% methylene green and mounted in aqueous based media. Microscopy was conducted with an Olympus BX46 light microscope and captured with the Olympus DP21 digital camera.

### RNA ISH image analysis and quantification

Images of tissue sections were analyzed by our in-house image processing package (imQui), which provided an interactive, user-interface to test and setup the processing pipeline as well as the utility to execute the developed pipeline on the set of images. First, each color image was separated into its red, green, and blue components. As the red channel image contained the least noise among the three colors, it was chosen for nuclei segmentation and measurements. The red image was inverted to appear like a fluorescence image, such that previously developed nuclei segmentation algorithms could be used [46]. Background subtraction was employed to compensate for lighting and shading variations in the image. Otsu thresholding and watershed segmentation were then applied to detect and separate any touching nuclei. For each nuclear defined region, the mean intensity of the object (in the red channel image) was evaluated. Finally, the estimated absorbance density for each object was evaluated by subtracting the logarithm of the mean object intensity by the logarithm of the illumination intensity (the same illumination intensity level was used for all images, since the same imaging conditions were applied to all images). Comparison of significance in differences between samples was performed using a two-sided Student's t-test on the optical density values.

### Affymetrix and QPCR statistical analysis

(i) Affymetrix chip data was quantile normalized and corrected using the GC-RMA algorithm [47]. Stratagene ArrayAssist version 5.1.0 was used to analyze the Affymetrix data (located at: http://www.ncbi.nlm.nih.gov/geo/query/acc.cgi?token=vxytbewkkqaqeze&acc=GSE28383) and create volcano plots. Experimental groups were compared using unpaired t-tests. Differential expression was analyzed with gene-level and exon level models in ArrayAssist and genes passing the filters (Figure 1) of > 1.5 fold expression difference and *p *< 0.05 after correcting for multiple comparisons were included. Using the volcano plot distributions additional outliers (locally isolated data points, showing large fold change or small *p*-value, Additional file 1) were included. Table S1 in Additional file 5 lists the characteristics and probe values for the 198 genes passing these criteria.

(ii) QPCR data was analyzed using linear mixed effects models, with experimental groups compared via the likelihood ratio chi-square statistic [48,49]. Adjustment for multiple comparisons was performed using the Benjamini-Hochberg method [50]. For the testosterone challenge experiment, a linear mixed effects model was used to relate raw Ct values from loading control genes (*Gapdh*, 18S rRNA) and test genes.(1.1)

*Y_ijklm _*= QPCR cycle time for gene target i, genotype j, hormone condition k, animal l(jk) and technical replicate m(ijkl) where;

i = 1 (loading control), 2 (gene of interest)

j = 1 (WT), 2 (KO)

k = 1 (No testosterone in implant), 2 (testosterone in implant)

l = 1, 2, ..., n_l(jk) _indicates mouse number for conditions j and k

m = 1, 2, ..., n_m(ijkl) _indicates technical replicate for conditions i, j, k, l.

μ = overall Ct level

δ_*i *_= change in Ct level corresponding to gene target (loading control or gene of interest)

γ_*j *_= change in Ct level due to genotype

τ_*k *_= change in Ct level due to hormone implant

α_*l*(*jk*) _= random effect on Ct level attributable to the lth animal within conditions j and k, arising from a Gaussian distribution with mean 0 and variance σ_α_

ε_*m*(*ijkl*) _= random effect on Ct level due to technical variability, arising from a Gaussian distribution with mean 0 and variance σ_ε_

The remaining terms δγ_*ij*_, δτ_*ik*_, δτ_*jk *_and δγτ_*ijk *_represent second and third order interaction terms of the three main effects. Estimates of these parameters obtained from the data via restricted maximum likelihood fitting (REML) will be denoted with a circumflex, for example the estimate of the difference in expression level due to genotype *j *γ_*j *_is denoted as .

δγ_*ij *_allows the model to reflect a different expression level in the gene of interest in the KO (Δ*C*_*t *_in the KO) as compared to that seen in the WT in the placebo implanted animals (Δ*C*_*t *_in the WT). Thus in QPCR terminology ΔΔ*C*_*t *_for the placebo implanted animals is estimated by .

δγτ_*ijk *_allows the model to reflect a different expression level in the gene of interest in the KO (Δ*C*_*t *_in the KO) as compared to that seen in the WT in the testosterone implanted animals (Δ*C*_*t *_in the WT). In QPCR terminology ΔΔ*C*_*t *_for the testosterone implanted animals is estimated by .

δτ_*ik *_allows the model reflect a different baseline expression level of the gene of interest in the testosterone implanted animals relative to the placebo implanted animals.

γτ_*jk *_allows the model to reflect a different baseline expression level of the loading control in the knockout animals relative to the wild-type animals.

In the terminology of linear mixed effects models, gene target, genotype and hormone implant factors are fixed effect factors; and animal is a random effects factor. Animal is modeled as a random effect as the animal effect for each animal is not of interest in this experiment. Rather, the results are to be interpreted with respect to the overall mouse population, and the animals included in this experiment represent a random sample from the overall population. The two genotypes, *Kiss1 *knockout and *Gpr54 *knockout, were modeled separately using the model (1.1). Although all factors could have been put in one 2 × 2 × 3 model, for simplicity the data from *Kiss1 *and *Gpr54 *knockouts were modeled separately, in two 2 × 2 × 2 factorial designs (genotype: wt or ko; treatment: T- or T+; gene: test or loading control). The relationship between the model-estimated means for each of the conditions allowed inference of four effect types of interest. These were: genotype only transcription differences, hormone treatment only transcription differences, hormone and genotype differences where the rate of difference is symmetrical (no interaction between effects), and hormone and genotype differences where the differences between groups show an effect interaction (ie the difference for hormone treatment is disproportionately greater in one genotype). The linear model was fitted using the lmer function from the lme4 package implemented in R-2.7.0 [51,52]. The model output was used to construct estimates of the mean relative effect and direction, 95% confidence intervals, and omnibus *p*-value (from likelihood-ratio chi-square test of any effect due to genotype or hormone adjusted for multiple corrections by the Benjamini Hochberg method).

## Authors' contributions

LMP performed all experiments except the murine studies, kisspeptin IHC, and TMEM144 western blot, drafted the original manuscript, and participated in manuscript editing. XD performed all murine studies except for the mice used in the NPAS4 IHC and *Tmem*144 ISH analysis, performed the testosterone assay, performed the kisspeptin IHC and TMEM144 western blot, and participated in manuscript editing. SM performed all the statistical analysis for the Affymetrix and real-time PCR and participated in manuscript editing. TR maintained the mouse husbandry and performed the brain extractions for the NPAS4 IHC and *Tmem144 *ISH studies. DY assisted in establishing the ISH protocol and ISH experiment. GT quantified the IHC expression. SP quantified the ISH expression. MS participated in the murine IHC and ISH studies. PA performed the brain sectioning for the IHC and ISH studies. AB performed the microscopic photography for the IHC studies. JF participated in the initial experiments. DGH participated in the study design and manuscript editing. SAJA performed the network analysis, and both SAJA and WHC conceived of the study design and led the manuscript editing. All authors have read and approve of the final manuscript.

## Supplementary Material

Additional file 1 **Supplemental Figure 1. Volcano plots from the Affymetrix Exon 1.0 ST Array**. Genes that were considered for further analysis are represented by yellow squares. The x-axis is the fold change for comparison groups that are indicated on the left side of the figures. The y-axis is the *p*-value among biological replicates. Click here for file

Additional file 2 **Supplemental Figure 2. GKO hypothalamic transcription networks merged**. The top three networks of GKO hypothalamic transcription merged. Click here for file

Additional file 3 **Supplemental Figure 4. GKO and KKO hypothalamic transcription networks**. The top three networks for both GKO and KKO hypothalamic transcription shown separately. Click here for file

Additional file 4 **Supplemental Figure 3. KKO hypothalamic transcription networks merged**. The top three networks of KKO hypothalamic transcription merged. Click here for file

Additional file 5 **Supplemental Table 1**. The characteristics and probe locations of the outliers from the volcano plots representing the Affymetrix results. Click here for file

Additional file 6 **Supplemental Table 2. Genes carried forward for QPCR validation from initial array analysis**. Lists the 95 genes assessed by QPCR that were chosen from the initial array analysis. Click here for file

Additional file 7 **Supplemental Table 4. Characteristics of the hormonally treated mice**. Lists the characteristics of the mice used in the T response cohort. Click here for file

Additional file 8 **Supplemental Tables 5-12**. These tables list the complete model results for the four classes of variation for each genotype: (i) purely hormone-dependent transcription (ii) purely genotype-dependent transcription (iii) hormone and genotype-dependent transcription but with no interaction between these variables and (iv) hormone and genotype-dependent transcription, with co-dependence (interaction) between these variables. Click here for file

Additional file 9 **Supplemental Figure 5. *Tmem144 in situ *hybridization in the hypothalamus of WT and KKO male mice**. *Tmem144 in situ *hybridization in the ARC of the hypothalamus of WT (top) and KKO (bottom) intact male mice (A). The sense probe is used as a negative control and is shown on the left while the antisense probe stains as a dark brown precipitate and is shown on the right. Quantification of the optical density is shown in a bar graph (B) where the asterisk denotes statistical significance between the means. *: *p *> 0.01. S: sense. AS: antisense. 3 V: third ventricle. Scale bar represents 100 μm. Click here for file

Additional file 10 **Supplemental Figure 6. TMEM144 protein content in the hypothalamus of WT and KKO male mice**. Immunoblotting of anterior hypothalamus (A) or posterior hypothalamus (B) protein lysates showed bands at 39 kDa for TMEM144 and 50 kDa for β-tubulin. Bargraphs represent the TMEM144 protein levels in the anterior (A) and the posterior hypothalamus (B), and are expressed in arbitrary unit as the mean of the ratio Tmem144/β-tubulin in each sample. Note the significantly higher TMEM144 protein expression in the anterior hypothalamus from KKO mice (* *p *= 0.034, n = 4 for each). Individuals are numbered 1 to 8. Click here for file

Additional file 11 **Supplemental Table 3. Genes carried forward into the T response QPCR analysis**. Lists the 48 genes assessed by QPCR using the T response cohort of mice. Click here for file
