# Supplementary figures and images for: The testosterone-dependent and independent transcriptional networks in the hypothalamus of Gpr54 and Kiss1 knockout male mice are not fully equivalent
Source: BMC Genomics. 2011 Apr 28;12:209. doi: 10.1186/1471-2164-12-209 (PMC3111392; doi:10.1186/1471-2164-12-209)

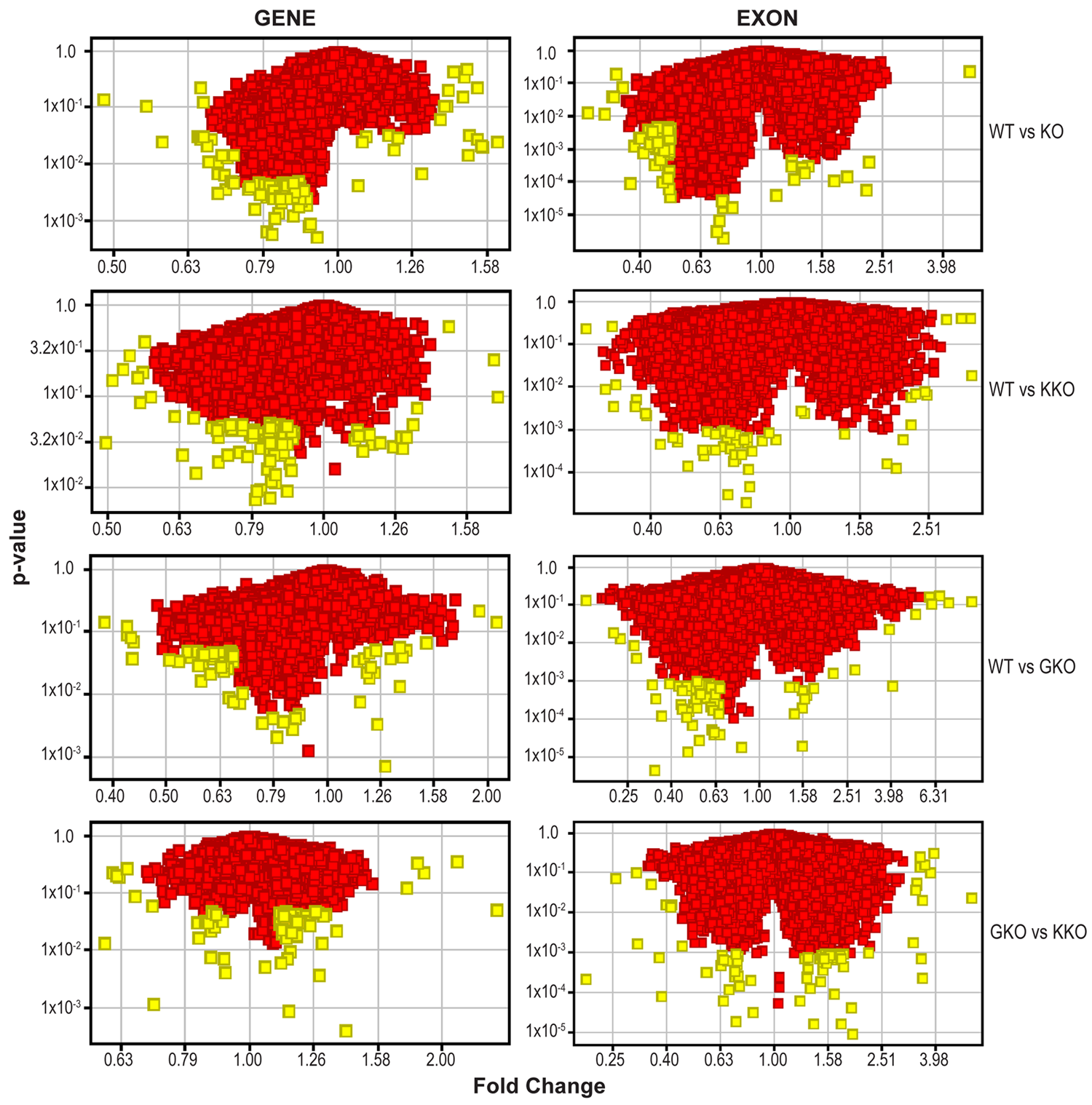

Supplement: Additional file 1 — Supplemental Figure 1. Volcano plots from the Affymetrix Exon 1.0 ST Array. Genes that were considered for further analysis are represented by yellow squares. The x-axis is the fold change for comparison groups that are indicated on the left side of the figures. The y-axis is the p-value among biological replicates. [file 1471-2164-12-209-S1.TIFF]

## GPR54 knockout hypothalamic transcription network (merged top 3 networks)

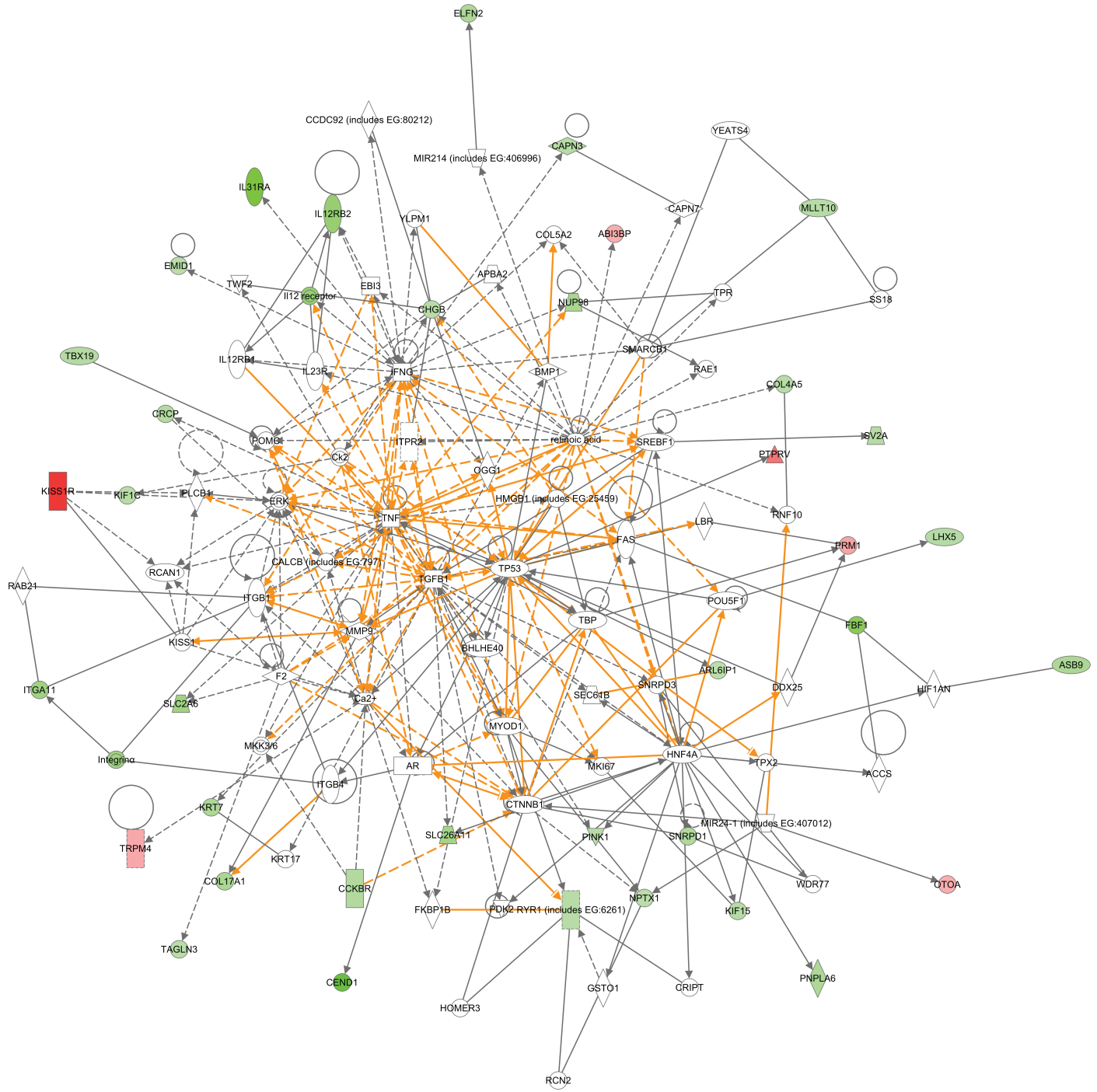

Supplement: Additional file 2 — Supplemental Figure 2. GKO hypothalamic transcription networks merged. The top three networks of GKO hypothalamic transcription merged. [file 1471-2164-12-209-S2.PDF]

# Kisspeptin knockout hypothalamic transcription networks (merged top 3 networks)

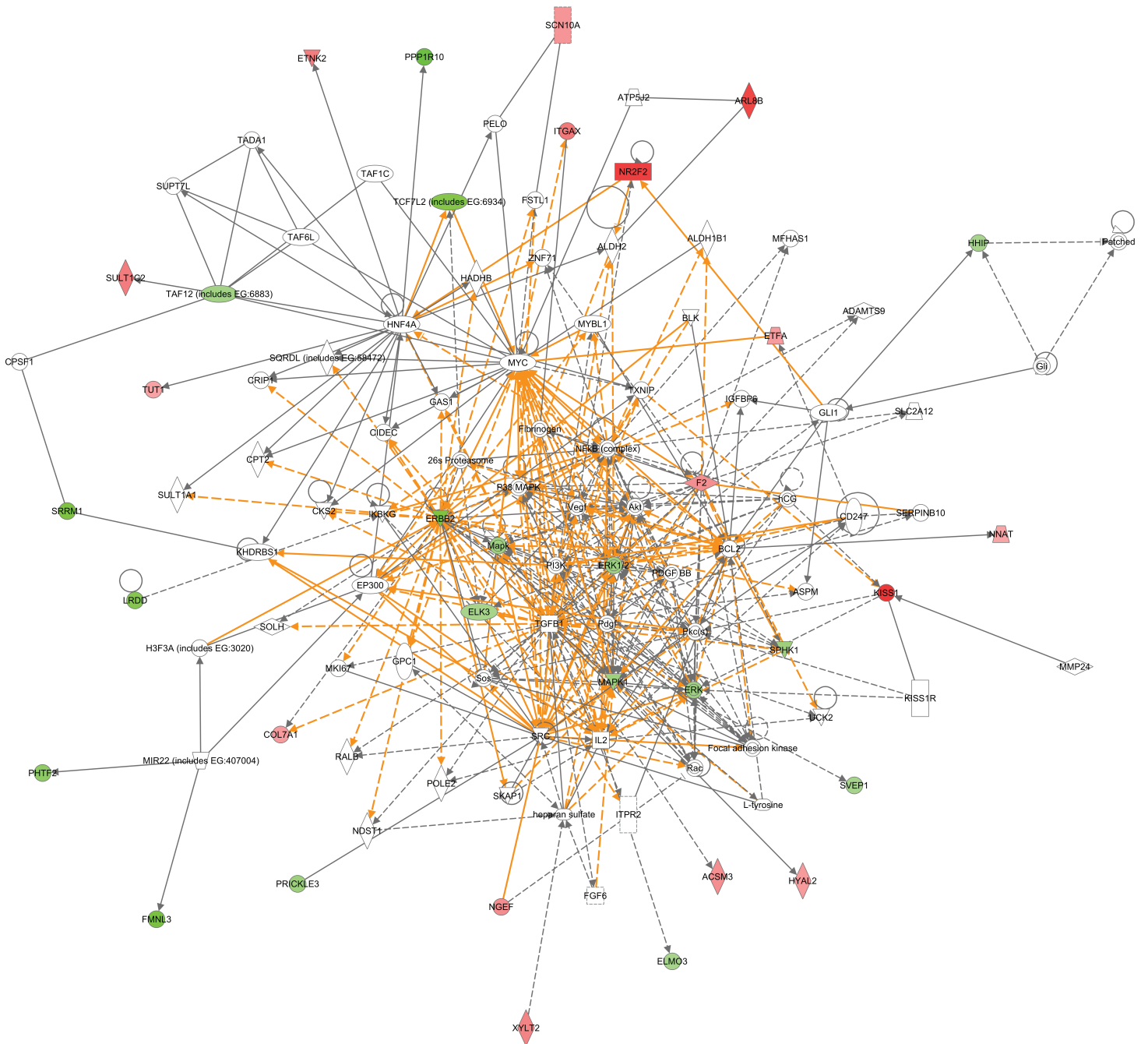

Supplement: Additional file 4 — Supplemental Figure 3. KKO hypothalamic transcription networks merged. The top three networks of KKO hypothalamic transcription merged. [file 1471-2164-12-209-S4.PDF]

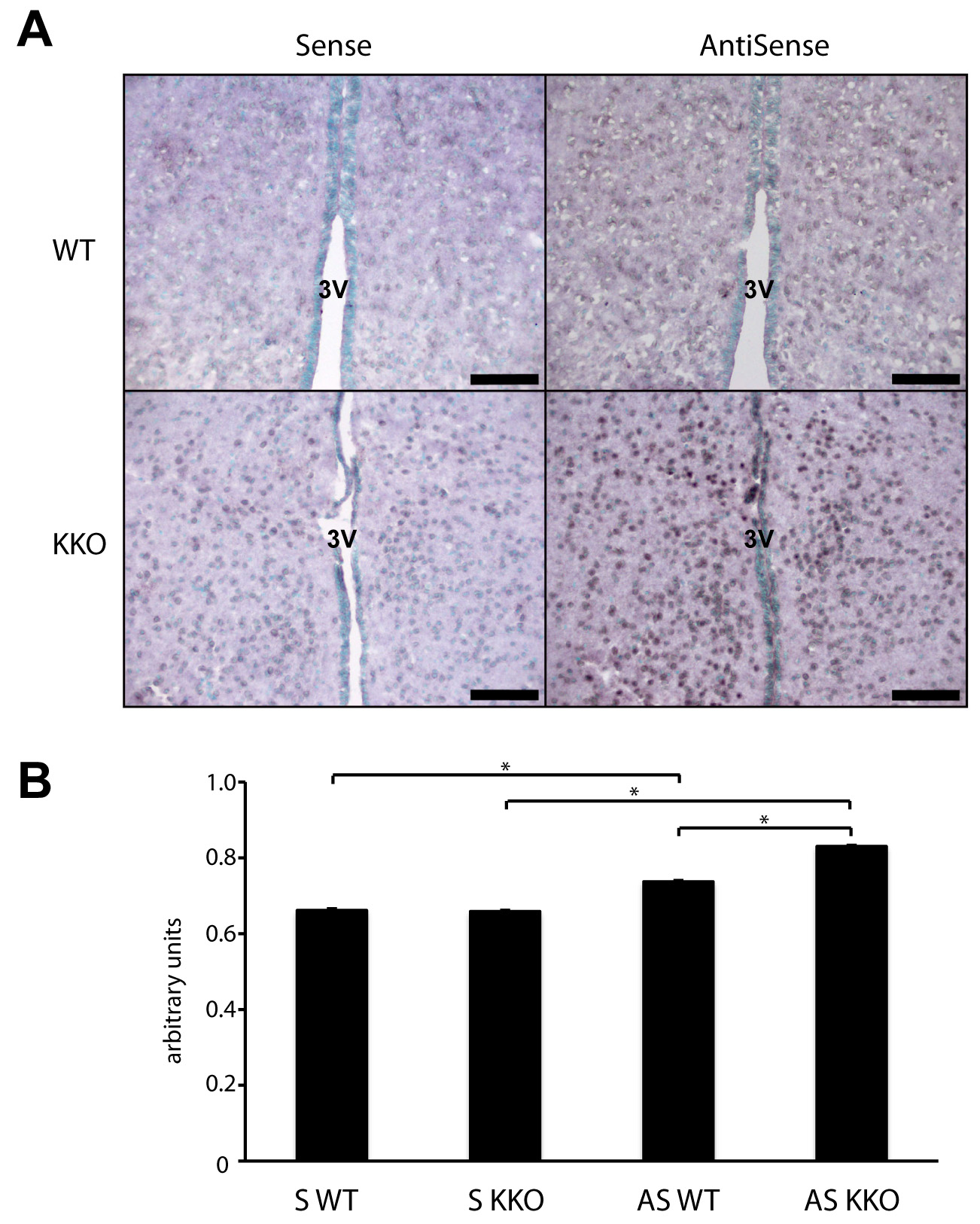

Supplement: Additional file 9 — Supplemental Figure 5. Tmem144 in situ hybridization in the hypothalamus of WT and KKO male mice. Tmem144 in situ hybridization in the ARC of the hypothalamus of WT (top) and KKO (bottom) intact male mice (A). The sense probe is used as a negative control and is shown on the left while the antisense probe stains as a dark brown precipitate and is shown on the right. Quantification of the optical density is shown in a bar graph (B) where the asterisk denotes statistical significance between the means. *: p > 0.01. S: sense. AS: antisense. 3 V: third ventricle. Scale bar represents 100 μm. [file 1471-2164-12-209-S9.JPEG]

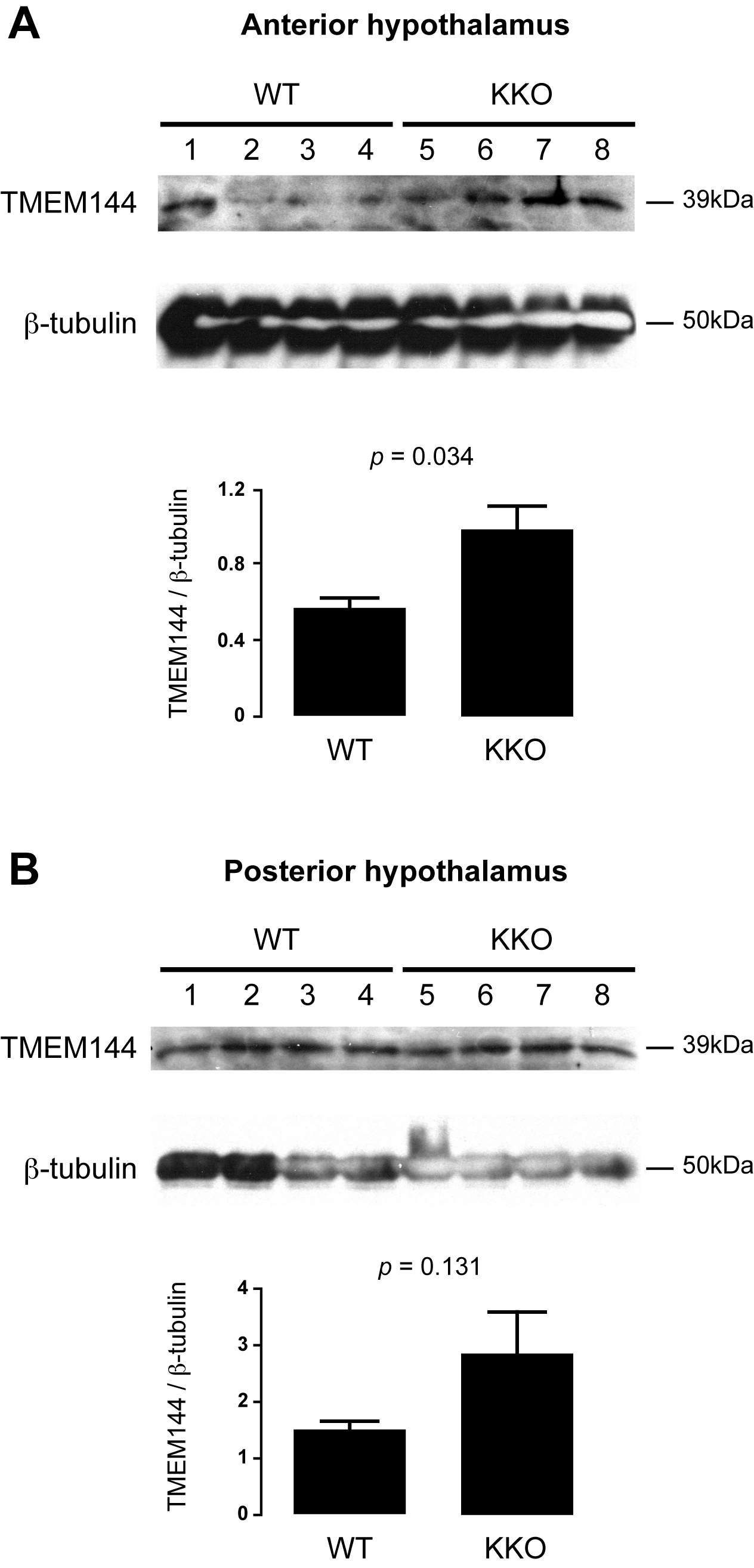

Supplement: Additional file 10 — Supplemental Figure 6. TMEM144 protein content in the hypothalamus of WT and KKO male mice. Immunoblotting of anterior hypothalamus (A) or posterior hypothalamus (B) protein lysates showed bands at 39 kDa for TMEM144 and 50 kDa for β-tubulin. Bargraphs represent the TMEM144 protein levels in the anterior (A) and the posterior hypothalamus (B), and are expressed in arbitrary unit as the mean of the ratio Tmem144/β-tubulin in each sample. Note the significantly higher TMEM144 protein expression in the anterior hypothalamus from KKO mice (* p = 0.034, n = 4 for each). Individuals are numbered 1 to 8. [file 1471-2164-12-209-S10.JPEG]
