# Supplementary material for: The testosterone-dependent and independent transcriptional networks in the hypothalamus of Gpr54 and Kiss1 knockout male mice are not fully equivalent
Source: BMC Genomics. 2011 Apr 28;12:209. doi: 10.1186/1471-2164-12-209 (PMC3111392; doi:10.1186/1471-2164-12-209)

Kisspeptin knockout hypothalamus transcript networks

GPR54 knockout hypothalamus transcript networks

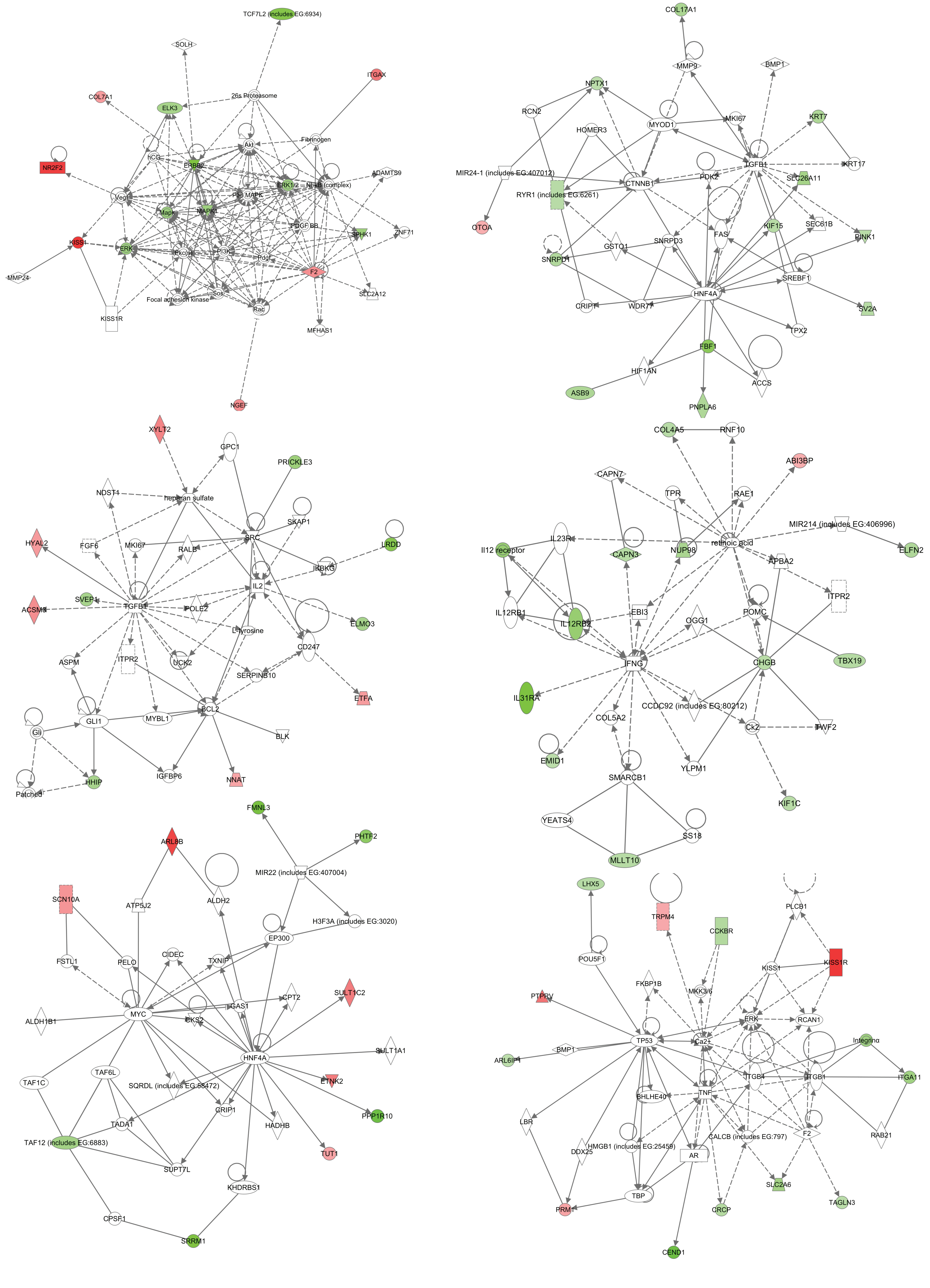

Supplement: Additional file 3 — Supplemental Figure 4. GKO and KKO hypothalamic transcription networks. The top three networks for both GKO and KKO hypothalamic transcription shown separately. [file 1471-2164-12-209-S3.PDF]
