# Supplementary material for: The testosterone-dependent and independent transcriptional networks in the hypothalamus of Gpr54 and Kiss1 knockout male mice are not fully equivalent
Source: BMC Genomics. 2011 Apr 28;12:209. doi: 10.1186/1471-2164-12-209 (PMC3111392; doi:10.1186/1471-2164-12-209)
Supplement: Additional file 6 — Supplemental Table 2. Genes carried forward for QPCR validation from initial array analysis. Lists the 95 genes assessed by QPCR that were chosen from the initial array analysis. [file 1471-2164-12-209-S6.PDF]

Supplemental Table 2. Genes carried forward for QPCR validation from initial array analysis

Gene	Assay ID	Gene	Assay ID	Gene	Assay ID
**18S**	**Hs99999901_s1**	*Htr2c*	Mm00434127_m1	***Pdyn***	**Mm00457572_m1**
Abca8a	Mm00462440_m1	*Hyal2*	Mm00477731_m1	*Pgm1*	Mm00804141_m1
Acsm3	Mm00489774_m1	*Il15ra*	Mm00500457_m1	***Pgr***	**Mm00435625_m1**
Adamtsl4	Mm00523242_m1	*Il8ra*	Mm00731329_s1	*Phtf2*	Mm00557065_m1
Ar	**Mm00442688_m1**	*Irak3*	Mm00518541_m1	*Ppef2*	Mm00448366_m1
Boc	Mm00552900_m1	*Itgax*	Mm00498698_m1	*Prickle3*	Mm00558283_m1
Centd2	Mm00546699_g1	*Itgbl1*	Mm00520942_m1	*Pus7*	Mm00617871_m1
Cln3	Mm00487021_m1	*Itpr3*	Mm00446540_m1	*Rab34*	Mm00446343_g1
Col7a1	Mm00483818_m1	***Kiss1***	**Mm00617576_m1**	*Rragd*	Mm00546741_m1
Ddx3y	Mm00465349_m1	***Kiss1r***	**Mm00475046_m1**	*Sh3tc2*	Mm00553970_m1
Dmn	Mm00809202_s1	*Krit1*	Mm00459502_m1	*Six2*	Mm00807058_m1
Dmrt3	Mm00616649_m1	***Lep***	**Mm00434759_m1**	*Slc25a25*	Mm00525104_m1
Eda	Mm00438653_m1	***Lepr***	**Mm00440174_m1**	*Slc26a8*	Mm00524836_m1
Edc4	Mm00725090_m1	***Lhb***	**Mm00656868_g1**	***Sp1***	**Mm00489039_m1**
Eif2s3y	Mm00468995_g1	***Lhcgr***	**Mm00442931_m1**	*Speer4b*	Mm00835620_m1
Elk3	Mm00469054_m1	*Lrdd*	Mm00502614_m1	*Sphk1*	Mm01252544_m1
Elmo3	Mm00555221_g1	*Mapk1*	Mm00442479_m1	*Srpk3*	Mm00444746_m1
Ermap	Mm00469273_m1	***Med23***	**Mm00518410_m1**	*Srrm1*	Mm00489728_m1
Ero1l	Mm00469296_m1	*Metapl1*	Mm00491809_m1	*Sult1c2*	Mm00471845_m1
Esr1	**Mm00433149_m1**	***Mmp2***	**Mm00439506_m1**	*Svep1*	Mm00465696_m1
Esr2	**Mm00599819_m1**	*Mmp28*	Mm00712992_m1	*Tac2*	Mm00436885_m1
Fsd2	Mm00556634_m1	***Mmp9***	**Mm00442991_m1**	***Tacr3***	**Mm00445346_m1**
Fzd10	Mm00558396_s1	*Mobkl2c*	Mm00774347_m1	*Taf12*	Mm00499416_m1
Gapdh	**Mm99999915_g1**	*Myh9*	Mm00502575_m1	*Tcf7l2*	Mm00501505_m1
Glis3	Mm00615386_m1	*Ngef*	Mm00451232_m1	*Tec*	Mm00443230_m1
Gnrhr	**Mm00439143_m1**	*Npas4*	Mm00463644_m1	*Tmem144*	Mm00510477_m1
Goltla	**Mm00503319_m1**	***Npy***	**Mm00445771_m1**	***Txnip***	**Mm00452393_m1**
Gpr143	Mm00440553_m1	*Nr2f2*	Mm00772789_m1	*Ucn*	Mm00445261_m1
Gpr146	Mm01700739_m1	*Oas3*	Mm00460944_m1	***Wnt5a***	**Mm00437347_m1**
Gtf3a	Mm00550608_m1	*Olig2*	Mm01210556_m1	*Zfp472*	Mm00461969_m1
Hapln2	Mm00480745_m1	*Opn4*	Mm00443523_m1	*Zmym6*	Mm00624222_m1
Hhip	Mm00469580_m1	*Pbx2*	Mm00479560_m1		

*One position is empty on the part of the manufacturer

Genes in **bold** are controls or those of interest that were not observed in the initial affymetrix analysis.

Genes not bolded were derived from the initial array analysis.
