# Supplementary material for: The testosterone-dependent and independent transcriptional networks in the hypothalamus of Gpr54 and Kiss1 knockout male mice are not fully equivalent
Source: BMC Genomics. 2011 Apr 28;12:209. doi: 10.1186/1471-2164-12-209 (PMC3111392; doi:10.1186/1471-2164-12-209)
Supplement: Additional file 7 — Supplemental Table 4. Characteristics of the hormonally treated mice. Lists the characteristics of the mice used in the T response cohort. [file 1471-2164-12-209-S7.PDF]

Supplemental Table 4. Characteristics of the hormonally treated mice

Mouse ID	Genotype	Sex	Weight	Testosterone	Implant	Date of Birth	Date of Castration	Date of Sacrifice
KJ74	K-/-	Male	20 g	0 pg/mL	Empty	29/08/08	15/10/08	12/11/08
KJ59	K-/-	Male	21 g	0 pg/mL	Empty	17/08/08	15/10/08	12/11/08
KJ10	K-/-	Male	20 g	0 pg/mL	Empty	04/08/08	30/09/08	21/10/08
KJ25	K-/-	Male	20 g	5.7 pg/mL	Testosterone	25/07/08	30/09/08	21/10/08
KJ55	K-/-	Male	21 g	2.8 pg/mL	Testosterone	15/08/08	15/10/08	12/11/08
KJ47	K-/-	Male	22 g	3.4 pg/mL	Testosterone	14/08/08	15/10/08	12/11/08
HL73	G-/-	Male	21 g	0 pg/mL	Empty	18/08/08	15/10/08	12/11/08
HL74	G-/-	Male	21 g	0 pg/mL	Empty	18/08/08	15/10/08	12/11/08
HL27	G-/-	Male	20 g	0 pg/mL	Empty	19/07/08	30/09/08	21/10/08
HL61	G-/-	Male	17 g	5.1 pg/mL	Testosterone	24/08/08	15/10/08	12/11/08
HK97	G-/-	Male	23 g	8.8 pg/mL	Testosterone	07/07/08	30/09/08	21/10/08
HL49	G-/-	Male	22 g	3.6 pg/mL	Testosterone	25/07/08	30/09/08	21/10/08
KJ52	WT+/+	Male	21 g	0 pg/mL	Empty	14/08/08	15/10/08	12/11/08
KJ64	WT+/+	Male	29 g	0 pg/mL	Empty	19/08/08	15/10/08	12/11/08
HL31	WT+/+	Male	28 g	0 pg/mL	Empty	19/07/08	30/09/08	21/10/08
HL30	WT+/+	Male	26 g	4.2 pg/mL	Testosterone	19/07/08	30/09/08	21/10/08
HK99	WT+/+	Male	27 g	4.8 pg/mL	Testosterone	07/07/08	30/09/08	21/10/08
KJ46	WT+/+	Male	24 g	7 pg/mL	Testosterone	14/08/08	15/10/08	12/11/08
