# Supplementary material for: The testosterone-dependent and independent transcriptional networks in the hypothalamus of Gpr54 and Kiss1 knockout male mice are not fully equivalent
Source: BMC Genomics. 2011 Apr 28;12:209. doi: 10.1186/1471-2164-12-209 (PMC3111392; doi:10.1186/1471-2164-12-209)
Supplement: Additional file 8 — Supplemental Tables 5-12. These tables list the complete model results for the four classes of variation for each genotype: (i) purely hormone-dependent transcription (ii) purely genotype-dependent transcription (iii) hormone and genotype-dependent transcription but with no interaction between these variables and (iv) hormone and genotype-dependent transcription, with co-dependence (interaction) between these variables. [file 1471-2164-12-209-S8.PDF]

Supplemental Table 5. KKO hormone only effect genes compared with testosterone treatment

	Hormonal Effect		
Gene	FC (95%CI)	Direction	p-value
Esr1	1.23(1.14-1.32)	Down	0.0003
Gnrhr	2.33(1.63-3.33)	Down	0.0009

Supplemental Table 6. KKO genotype effect only genes compared with WT

	Genotype Effect		
Gene	FC (95%CI)	Direction	p-value
Ar	1.16(1.08-1.25)	Down	0.0272
Eif2s3y	1.27(1.09-1.48)	Down	0.0225
Tmem144	1.50(1.30-1.73)	Up	6.12E-06

Supplemental Table 7. KKO hormone and genotype effect non-interacting genes

	Genotype Effect		Hormonal Effect		
Gene	FC (95%CI)	Direction	FC (95%CI)	Direction	p-value
Abca8a	1.21(1.06-1.37)	Up	1.64(1.45-1.86)	Down	1.75E-09
Ddx3y	1.18(1.06-1.32)	Down	1.25(1.12-1.39)	Down	0.0013
Fzd10	1.16(1.03-1.31)	Up	1.18(1.05-1.33)	Down	0.0272

Supplemental Table 8. KKO hormone and genotype effect interacting genes

	Genotype Effect within Hormonal Group				Hormonal Effect within Genotype				
	No testosterone		Testosterone		Wiltype		Knockout		
Gene	FC (95%CI)	Dir	FC (95%CI)	Dir	FC (95%CI)	Dir	FC (95%CI)	Dir	p-value
Acsm3	1.23(1.03-1.48)	Up	1.27(1.03-1.56)	Down	1.86(1.53-2.26)	Up	1.19(0.98-1.45)	Up	9.80E-06
Klk1b22	1.30(0.92-1.84)	Down	4.30(3.01-6.15)	Down	1.34(0.97-1.84)	Down	4.43(3.01-6.51)	Down	1.75E-09
Mmp9	1.03(0.68-1.56)	Down	3.75(2.44-5.75)	Down	1.08(0.74-1.59)	Down	3.93(2.48-6.22)	Down	0.0127
Six2	1.08(0.84-1.40)	Up	1.99(1.54-2.57)	Up	1.60(1.24-2.07)	Down	1.15(0.89-1.49)	Up	1.14E-05
Tec	1.07(0.86-1.32)	Down	1.72(1.39-2.12)	Up	1.63(1.32-2.02)	Down	1.12(0.91-1.38)	Up	1.82E-05
Txnip	1.16(1.00-1.34)	Down	1.22(1.06-1.41)	Up	1.66(1.44-1.92)	Down	1.18(1.02-1.36)	Down	3.01E-08

Supplemental Table 9. GKO hormone only effect genes compared with testosterone treatment

Hormonal Effect			
**Gene**	**FC (95%CI)**	**Direction**	**p-value**
Abca8a	1.57(1.33-1.87)	Down	2.83E-05
Hyal2	1.13(1.05-1.22)	Down	0.0422
Klk1b22	2.16(1.43-3.25)	Down	0.0042
Mmp2	1.23(1.10-1.37)	Down	0.0113
Pgr	1.28(1.18-1.37)	Up	5.66E-07

Supplemental Table 10. GKO genotype effect only genes compared with WT

Genotype Effect			
**Gene**	**FC (95%CI)**	**Direction**	**p-value**
Arap1	1.21(1.10-1.34)	Down	0.0042
Boc	1.24(1.11-1.39)	Down	0.0136
Eif2s3y	1.48(1.25-1.74)	Down	0.0003
Ero1l	1.27(1.18-1.38)	Down	2.61E-05
Esr2	1.63(1.46-1.82)	Down	4.10E-10
Glis3	1.32(1.18-1.48)	Down	0.0004
Hapln2	1.43(1.28-1.60)	Down	2.61E-05
Hhip	1.63(1.46-1.82)	Down	4.68E-10
Htr2c	1.24(1.13-1.36)	Down	0.0003
Hyal2	1.25(1.13-1.39)	Down	0.0004
Lhcgr	1.61(1.30-1.99)	Down	0.0042
Lrdd	1.47(1.28-1.68)	Down	3.88E-05
Mmp28	1.48(1.25-1.73)	Down	0.0023
Mobkl2c	1.36(1.22-1.53)	Down	0.0004
Npas4	1.65(1.45-1.87)	Down	2.06E-08
Nr2f2	1.28(1.18-1.39)	Down	2.46E-05
Olig2	1.48(1.32-1.65)	Down	8.34E-07
Pgm1	1.26(1.15-1.37)	Down	0.0003
Phtf2	1.25(1.15-1.36)	Down	0.0001
Taf12	1.28(1.17-1.39)	Down	1.57E-05
Wnt5a	1.36(1.24-1.50)	Down	1.39E-06

Supplemental Table 11. GKO hormone and genotype effect non-interacting genes

	Genotype Effect		Hormonal Effect		
**Gene**	**FC (95%CI)**	**Direction**	**FC (95%CI)**	**Direction**	**p-value**
Abca8a	1.35(1.15-1.58)	Down	1.58(1.35-1.85)	Down	1.39E-07
Ddx3y	1.52(1.31-1.77)	Down	1.21(1.04-1.40)	Down	1.52E-05
Esr1	1.13(1.04-1.22)	Up	1.40(1.30-1.52)	Down	5.45E-10
Fzd10	1.36(1.20-1.53)	Down	1.16(1.03-1.31)	Down	6.00E-05
Kiss1	1.96(1.64-2.35)	Up	12.15(10.15-14.55)	Down	4.29E-46
Mmp2	1.41(1.27-1.56)	Down	1.23(1.12-1.36)	Down	2.06E-08
Pgr	1.28(1.18-1.38)	Down	1.27(1.18-1.37)	Down	1.51E-10
Tmem144	1.86(1.60-2.16)	Down	1.28(1.10-1.48)	Down	1.51E-10

Supplemental Table 12. GKO hormone and genotype effect interacting genes

	Genotype Effect within Hormonal Group				Hormonal Effect within Genotype				
	No testosterone		Testosterone		Wiltype		Knockout		
**Gene**	**FC (95%CI)**	**Dir**	**FC (95%CI)**	**Dir**	**FC (95%CI)**	**Dir**	**FC (95%CI)**	**Dir**	**p-value**
Acsm3	1.26(1.01-1.57)	Up	2.28(1.84-2.83)	Down	1.93(1.54-2.42)	Up	1.49(1.21-1.84)	Down	2.02E-08
Ar	1.23(1.09-1.38)	Down	1.56(1.39-1.75)	Down	1.13(1.01-1.27)	Up	1.12(1.00-1.25)	Down	1.02E-08
Ddx3y	1.30(1.13-1.50)	Down	1.01(0.88-1.16)	Up	1.38(1.20-1.59)	Down	1.05(0.91-1.20)	Down	0.0004
Eif2s3y	1.33(1.10-1.61)	Down	1.10(0.91-1.33)	Up	1.40(1.16-1.70)	Down	1.05(0.87-1.27)	Up	0.0196
Elk3	1.47(1.29-1.66)	Down	1.15(1.02-1.31)	Down	1.09(0.96-1.23)	Down	1.17(1.03-1.32)	Up	2.88E-06
Esr1	1.08(0.96-1.21)	Down	1.31(1.17-1.47)	Down	1.28(1.14-1.43)	Down	1.55(1.39-1.74)	Down	1.36E-11
Fzd10	1.20(1.01-1.42)	Down	1.17(0.99-1.40)	Up	1.37(1.15-1.63)	Down	1.02(0.86-1.22)	Up	0.0315
Gnrhr	1.28(0.78-2.12)	Down	3.20(1.70-6.03)	Up	2.29(1.37-3.82)	Down	1.80(0.96-3.35)	Up	1.80E-03
Kiss1	1.95(1.58-2.42)	Up	1.09(0.88-1.35)	Up	9.12(7.36-11.29)	Down	16.34(13.19-20.23)	Down	5.71E-52
Klk1b22	1.25(0.71-2.20)	Up	2.70(1.61-4.52)	Down	1.08(0.61-1.91)	Down	3.65(2.17-6.12)	Down	0.0004
Krit1	1.10(0.94-1.28)	Down	1.48(1.27-1.73)	Down	1.06(0.91-1.24)	Up	1.27(1.09-1.48)	Down	0.0005
Mmp9	1.11(0.91-1.35)	Up	1.70(1.40-2.07)	Up	1.11(0.92-1.35)	Up	1.71(1.41-2.08)	Up	2.69E-07
Ngef	1.09(0.97-1.23)	Down	1.44(1.27-1.62)	Down	1.15(1.01-1.29)	Up	1.15(1.02-1.30)	Down	5.16E-05
Six2	1.05(0.73-1.50)	Down	2.22(1.55-3.18)	Up	1.78(1.24-2.55)	Down	1.30(0.91-1.87)	Up	0.0022
Tec	2.48(1.95-3.14)	Down	1.75(1.38-2.22)	Down	1.63(1.29-2.07)	Down	1.16(0.91-1.46)	Down	2.03E-13
Txnip	1.04(0.80-1.34)	Down	1.81(1.40-2.35)	Up	1.85(1.43-2.39)	Down	1.02(0.79-1.32)	Up	8.79E-05
