# Supplementary material for: The testosterone-dependent and independent transcriptional networks in the hypothalamus of Gpr54 and Kiss1 knockout male mice are not fully equivalent
Source: BMC Genomics. 2011 Apr 28;12:209. doi: 10.1186/1471-2164-12-209 (PMC3111392; doi:10.1186/1471-2164-12-209)
Supplement: Additional file 11 — Supplemental Table 3. Genes carried forward into the T response QPCR analysis. Lists the 48 genes assessed by QPCR using the T response cohort of mice. [file 1471-2164-12-209-S11.PDF]

Supplemental Table 3. Genes carried forward into the T response QPCR analysis

48 Low Density Array

Gene	Assay ID	Gene	Assay ID
**18S**	**Hs99999901_s1**	*Klk1b22*	Mm02343755_g1
Abca8a	Mm00462440_m1	*Krit1*	Mm00459502_m1
Acsm3	Mm00489774_m1	***Lhcgr***	**Mm00442931_m1**
Ar	**Mm00442688_m1**	*Lrdd*	Mm00502614_m1
Arap1	Mm00546699_g1	***Mmp2***	**Mm00439508_m1**
Boc	Mm00552900_m1	*Mmp28*	Mm00712992_m1
Ddx3y	Mm00465349_m1	***Mmp9***	**Mm00442991_m1**
Eif2s3y	Mm00468995_g1	*Mobkl2c*	Mm00774347_m1
Elk3	Mm00469054_m1	*Ngef*	Mm00451232_m1
Ero1l	Mm00469296_m1	*Npas4*	Mm00463644_m1
Esr1	**Mm00433149_m1**	*Nr2f2*	Mm00772789_m1
Esr2	**Mm00599819_m1**	*Olig2*	Mm01210556_m1
Fzd10	Mm00558396_s1	*Pgm1*	Mm00804141_m1
Gapdh	**Mm99999915_g1**	***Pgr***	**Mm00435625_m1**
Glis3	Mm00615386_m1	*Phtf2*	Mm00557065_m1
Gnrhr	**Mm00439143_m1**	*Six2*	Mm00807058_m1
Hapln2	Mm00480745_m1	*Synm*	Mm00809202_s1
Hhip	Mm00469580_m1	*Tac2*	Mm00436885_m1
Htr2c	Mm00434127_m1	*Taf12*	Mm00499416_m1
Hyal2	Mm00477731_m1	*Tcf7l2*	Mm00501505_m1
Il15ra	Mm00500457_m1	*Tec*	Mm00443230_m1
Itgax	Mm00498698_m1	*Tmem144*	Mm00510477_m1
Kiss1	**Mm00617576_m1**	*Txnip*	Mm00452393_m1
Kiss1r	**Mm00475046_m1**	***Wnt5a***	**Mm00437347_m1**

Dmn=Synm, Centd2=Arap1

Genes in **bold** are controls or those of interest that were not observed in the initial affymetrix analysis.

Genes not bolded were derived from the initial array analysis.
